# Benchmarking large language models for extracting biobank-derived insights into health and disease

**DOI:** 10.1371/journal.pcbi.1014224

**Published:** 2026-04-20

**Authors:** Manuel Corpas, Alfredo Iacoangeli

**Affiliations:** 1 Life Sciences, University of Westminster, London, United Kingdom; 2 The Alan Turing Institute, London, United Kingdom; 3 Department of Biostatistics and Health Informatics, King’s College London, London, United Kingdom; 4 Department of Basic and Clinical Neuroscience, King’s College London, London, United Kingdom; 5 Perron Institute for Neurological and Translational Science, Perth, Western Australia, Australia; 6 Biomedical Research Centre, South London and Maudsley NHS Foundation Trust, London, United Kingdom; University of Maryland School of Medicine, UNITED STATES OF AMERICA

## Abstract

Biobank-scale datasets such as the UK Biobank have become foundational resources for advancing biomedical discovery. Yet the complexity and heterogeneity of these resources, spanning genomics, imaging, clinical records, and metadata, pose substantial barriers to access and interpretation. Large Language Models (LLMs) offer a promising avenue for making such datasets more navigable through natural language interfaces. However, the extent to which current general-purpose LLMs can retrieve and synthesize biobank-specific insights has not yet been systematically evaluated. In this study, we present a reproducible, multi-metric evaluation framework to benchmark the capabilities of leading LLMs. We evaluated six leading large language models: Gemini 3 Pro, Claude Opus 4.5, Claude Sonnet 4.5, GPT-5.2, Mistral Large 2, and DeepSeek V3, on four benchmark tasks designed to assess biobank-related knowledge retrieval. We evaluate model performance across six dimensions (semantic accuracy, factual correctness, domain knowledge, reasoning quality, response depth, and biobank specificity) and assessed output consistency using curated UK Biobank references and a robust random baseline. All models outperformed the baseline by 2× to 3× , with strong statistical separation (p < 0.001), confirming meaningful biobank-specific knowledge retrieval. Gemini 3 Pro achieved the highest overall accuracy across tasks such as keyword synthesis, institution recognition, and topic inference, while Claude Sonnet 4.5 demonstrated the most uniform performance across evaluation dimensions. Our benchmark provides a rigorous framework for evaluating LLMs in biomedical settings. Using the UK Biobank as a real-world testbed, we highlight both the capabilities and limitations of current models, measuring their capacity to recall structured biomedical knowledge consistent with authoritative biobank metadata.

## Author summary

The UK Biobank is one of the world’s most comprehensive biomedical datasets, containing detailed genetic, clinical, and lifestyle information from about 500,000 participants. While this resource has enabled thousands of studies, its complexity makes it difficult to access and interpret - especially for non-specialists or those outside of computational biology. Large Language Models (LLMs), such as ChatGPT, Claude, and Gemini, have emerged as promising tools to bridge this gap by offering intuitive, language-based access to scientific information. However, it remains unclear how effectively they can retrieve and contextualize the nuanced knowledge embedded in biobank-scale datasets. In this study, we benchmarked the performance of six frontier LLMs on tasks derived from UK Biobank metadata and literature. We developed a novel evaluation framework that measures not just keyword coverage, but also semantic accuracy, factual correctness, reasoning quality, and domain-specific understanding. Our results show that while all models performed significantly better than chance, only a few demonstrated consistent, high-fidelity understanding of biobank-specific content. This work provides a rigorous and reproducible method for evaluating LLM knowledge alignment in biomedical settings and highlights both the promise and current limitations of these models in retrieving and synthesizing biobank-relevant information.

## Introduction

Biobanks have become central to contemporary biomedical research, offering unprecedented scale and depth for studying population health, disease risk, and genotype-phenotype associations. Among them, the UK Biobank stands out as one of the most comprehensive and widely used resources, encompassing genomic, imaging, clinical, and lifestyle data from over 500,000 participants [1]. Its integration into thousands of scientific studies has made it a cornerstone of data-driven discovery in fields ranging from cardiometabolic diseases to neuropsychiatric traits [2–4].

Large Language Models (LLMs), with their ability to understand and generate human-like language [5], offer a potential means for literature mining, knowledge retrieval, and hypothesis generation [6]. However, while LLMs are increasingly used in biomedical applications, little is known about how well they perform in extracting or synthesizing information specific to biobank settings.

In this study, we address this gap by systematically benchmarking a set of leading LLMs, including GPT-5.2 [7], Claude Opus 4.5 and Claude Sonnet 4.5 [8], Gemini 3 Pro [9], Mistral Large 2 [10], and DeepSeek V3 [11], on tasks derived from the UK Biobank’s publications and application metadata. Our evaluation framework extends beyond keyword retrieval to encompass six dimensions: semantic accuracy, factual correctness, domain knowledge, reasoning quality, response depth, and biobank specificity. By benchmarking models across both structured and open-ended queries, and by systematically comparing their performance against a domain-specific random baseline, we capture not only their relative strengths and limitations but also the extent to which their outputs reflect meaningful, non-random knowledge synthesis. Improvement factors of 2× to 3× over random baselines, alongside statistically significant separation (p < 0.001), indicate that these LLMs retrieve biobank-relevant information with accuracy substantially exceeding chance, suggesting meaningful encoding of domain-specific knowledge during training.

## Methods

### Model selection and deployment strategy

To evaluate the capacity of general-purpose large language models (LLMs) to retrieve and synthesize biobank-related biomedical knowledge, we evaluated six leading large language models representing the current state-of-the-art as of January 2026. These models span a range of architectural families, training philosophies, and access paradigms. Included in our evaluation were Gemini 3 Pro (Google’s latest multimodal flagship model), Claude Opus 4.5 (Anthropic’s most capable reasoning model), Claude Sonnet 4.5 (Anthropic’s balanced performance model), GPT-5.2 (OpenAI’s latest generation model), Mistral Large 2 (Mistral AI’s flagship model), and DeepSeek V3 (DeepSeek’s latest general-purpose model).

All models were accessed via their official APIs in January 2026. Each model was queried with identical prompts, and responses were collected without fine-tuning or custom system prompts to ensure fair comparison of base model capabilities. This allowed us to benchmark each model’s general capability without the influence of task-specific optimization, reflecting how biomedical users typically engage with these systems in applied contexts.

To ensure comparability, each model was exposed to the same four queries, presented in identical phrasing and order. Responses were timestamped, archived in raw text and CSV format, and subsequently used for performance evaluation. Across all models, the integrity of the input-output pipeline was maintained to eliminate variability due to interface differences or prompt engineering.

### Reference corpora and ground truth curation

All evaluation tasks were anchored in data derived from the UK Biobank’s openly available metadata schemas [12]. Two key sources were used to construct domain-specific ground truth:

**Schema 19** provided a corpus of 8,553 publications that utilized UK Biobank data, of which 7,932 included an abstract. These publications span a wide range of biomedical domains, including cardiovascular, metabolic, neurological, and psychiatric research. We parsed this collection to extract the most frequent biomedical keywords, methodological terms, and phenotype descriptors. These included concepts such as “genome-wide association study,” “cardiovascular disease,” “prospective cohort,” and “Mendelian randomization.” Each concept was manually reviewed and mapped to a set of synonyms and lexical variants, enabling flexible matching to LLM outputs regardless of exact surface form.

**Schema 27** contained metadata from 15,046 approved UK Biobank research applications. This dataset included investigator names, project titles, and institutional affiliations. From this corpus, we extracted the top 20 most prolific authors and top 10 most active institutions as reference standards for the authorship and institutional retrieval tasks, respectively.

### Prompting and query standardization

To evaluate the models across a range of biomedical retrieval tasks, we formulated four standardized prompts reflecting common types of biobank-related queries. These were selected to span different forms of factual and inferential reasoning:

1. **Keyword Synthesis:** “What is the subject of the most commonly occurring keywords in UK Biobank papers?”
2. **Citation-Aware Topic Retrieval:** “What is the subject of the most cited papers relating to the UK Biobank?”
3. **Author Identification:** “Who are the top 20 most prolific authors publishing on the UK Biobank?”
4. **Institutional Recognition:** “What are the top 10 leading institutions in terms of number of applications to the UK Biobank?”

The four benchmark questions were selected to represent complementary dimensions of biobank-related information retrieval tasks that researchers commonly perform. Specifically: (1) Keyword Synthesis (Q1) tests the model’s ability to aggregate and summarize thematic content across a large corpus, a fundamental task in literature review and trend identification. (2) Citation-Aware Topic Retrieval (Q2) requires integrating bibliometric signals (citation counts) with content understanding, reflecting how researchers prioritize impactful literature. (3) Author Identification (Q3) evaluates entity recognition and ranking capabilities for person names, essential for identifying collaboration opportunities and key contributors to a field. (4) Institutional Recognition (Q4) assesses the model’s ability to extract and rank organizational entities from application metadata, which is critical for understanding the research landscape and institutional engagement patterns. These questions span keyword analysis, bibliometric integration, person entity extraction, and organizational entity extraction, four distinct competencies that collectively represent the range of information needs in biobank research discovery. This multi-faceted approach ensures the benchmark evaluates both factual recall and inferential synthesis across different entity types and data sources.

These prompts were designed to be deliberately open-ended, requiring each model not only to retrieve information, but to prioritize relevance and, ideally, demonstrate some capacity for synthesis. In the case of the keyword and citation questions, high-performing models were expected to surface core biomedical themes; for the authorship and institution questions, success required access to correct entities and some factual grounding.

Each prompt was submitted to every model in an isolated context without any conversational history. Responses were automatically extracted and stored for scoring. When responses included bullet points, tables, or summaries, we parsed these structures to preserve semantic content during subsequent evaluation.

### Scoring metrics and semantic matching framework

We developed a multi-tiered evaluation strategy that integrates both lexical and semantic similarity. Our primary metrics were Coverage Score, which measures the breadth of relevant concepts retrieved, and Weighted Coverage Score, which accounts for the frequency and salience of those concepts within the biobank literature.

To compute these metrics, we used the *all-MiniLM-L6-v2* SentenceTransformer model to encode both reference terms and model-generated outputs into vector embeddings. Cosine similarity was calculated between all term–response pairs, with a match recorded when similarity exceeded a threshold of 0.20. This threshold was chosen based on empirical tuning to balance precision and recall, and ambiguous matches were manually reviewed to ensure semantic validity.

**Fig 1:**
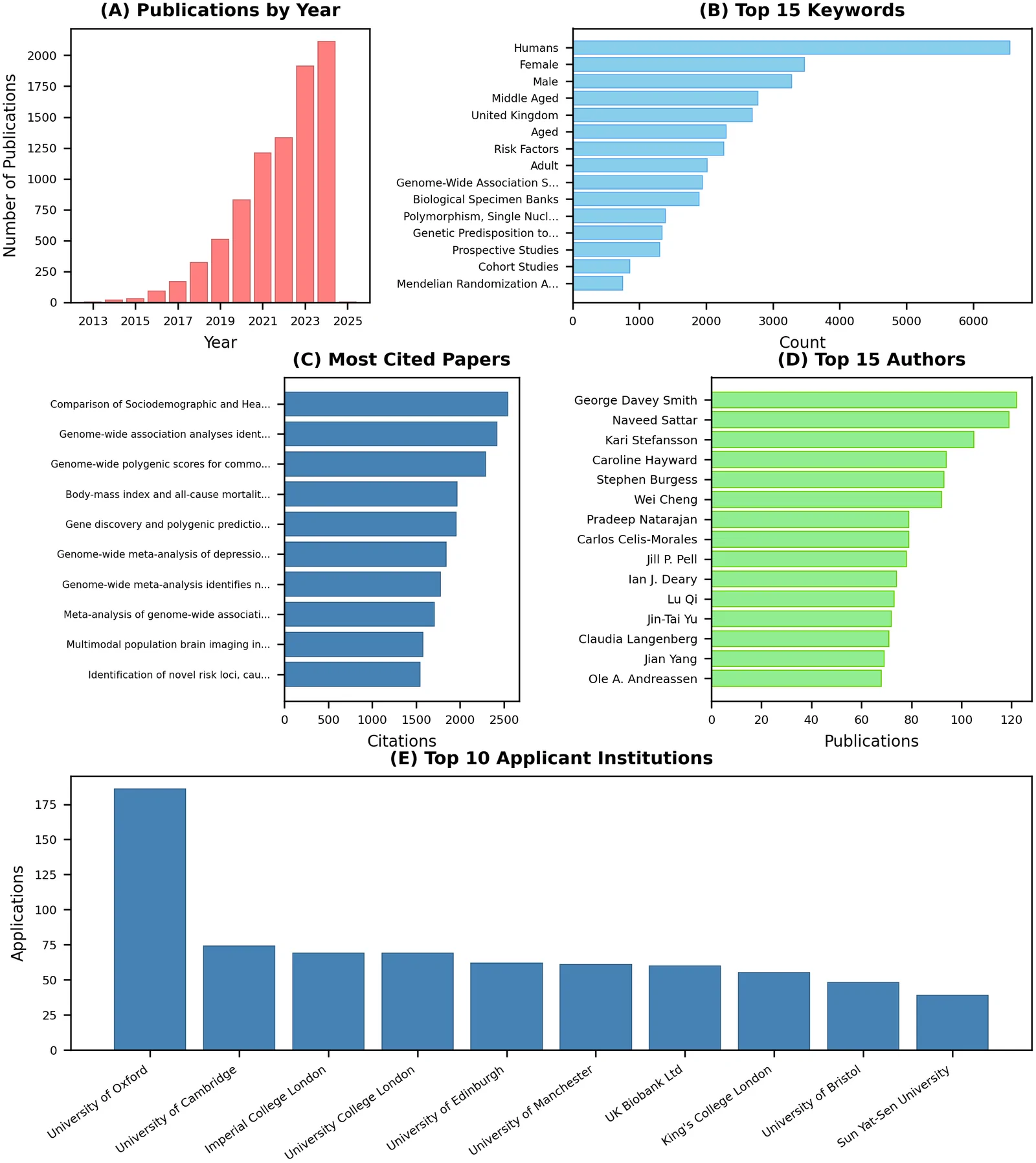
Benchmarking Results from UK Biobank Schema Data. **(A)** Publications by Year: Annual count of UK Biobank publications from Schema 19 (8,553 publications, of which 7,932 include an abstract; the chart counts all publications), showing growth from 2013 to 2025. **(B)** Top 15 Keywords: The most frequently cited keywords in UK Biobank papers, with “Humans” (6,547 occurrences) and demographic terms dominating, followed by methodological terms such as GWAS and Mendelian randomization. **(C)** Most Cited Papers: The ten most cited publications associated with the UK Biobank, with citation counts from Schema 19 metadata. Paper titles are truncated for display. **(D)** Top 15 Authors: The most prolific authors by publication count (e.g., George Davey Smith, 122 publications; Naveed Sattar, 119). **(E)** Top 10 Applicant Institutions: Leading institutions by number of approved UK Biobank research applications (Schema 27; 15,046 applications), with the University of Oxford (186 applications) ranking first.

The Weighted Coverage Score further incorporated frequency weights from Schema 19, assigning greater importance to high-impact terms such as “genome-wide association study,” which appears over 1,900 times (Fig 1B). This allowed us to prioritize core biomedical themes over peripheral mentions. These metrics quantify not only how much relevant content each model retrieved, but also how central that content is to UK Biobank research.

### Multidimensional evaluation of model capabilities

To assess interpretive competence beyond keyword retrieval, we developed a modular evaluation framework spanning six dimensions of large language model (LLM) performance on biobank-specific tasks. These dimensions were designed to capture semantic fidelity, reasoning depth, and domain-specific expertise relevant to biomedical research. All scores were calculated using custom Python scripts available in the project repository.

- **Semantic Accuracy:** Assessed whether retrieved biomedical concepts were not only semantically relevant but also appropriately contextualized. This score combined embedding-based cosine similarity (using SentenceTransformers) with expert manual review. Only correctly used biomedical concepts contributed to the final score.
- **Factual Correctness:** Evaluated entity-level precision in author and institution recognition by calculating the fraction of returned names matching those in the UK Biobank’s Schema 27 metadata. Only verifiable matches influenced this score.
- **Reasoning Quality:** Scored based on the presence of interpretive statements, causal relationships, or thematic synthesis, as opposed to flat or unordered lists. Responses exhibiting inference and contextual reasoning received higher ratings.
- **Domain Knowledge:** Measured both the frequency and correct usage of advanced biomedical concepts (e.g., *polygenic score*, *longitudinal analysis*, *Mendelian randomization*), including whether they were explained clearly and used appropriately.
- **Response Depth:** Captured whether responses demonstrated layered insight by integrating multiple aspects of biobank-related evidence (e.g., linking methodological approaches to disease categories). Shallow or unstructured responses were penalized.
- **Biobank Specificity:** Quantified the degree to which outputs were explicitly grounded in UK Biobank content. General biomedical responses that lacked biobank-specific anchoring received lower scores.

Detailed scoring rubrics for each evaluation dimension are provided in S1 Table (Supporting Information).

To assess performance stability, we calculated a Consistency Score, defined as a composite of the standard deviation and min-max range across all six metrics for a given model. Lower variability indicated more reliable performance across task types. These distributions are visualized in Fig 3E alongside semantic accuracy scores.

**Fig 2:**
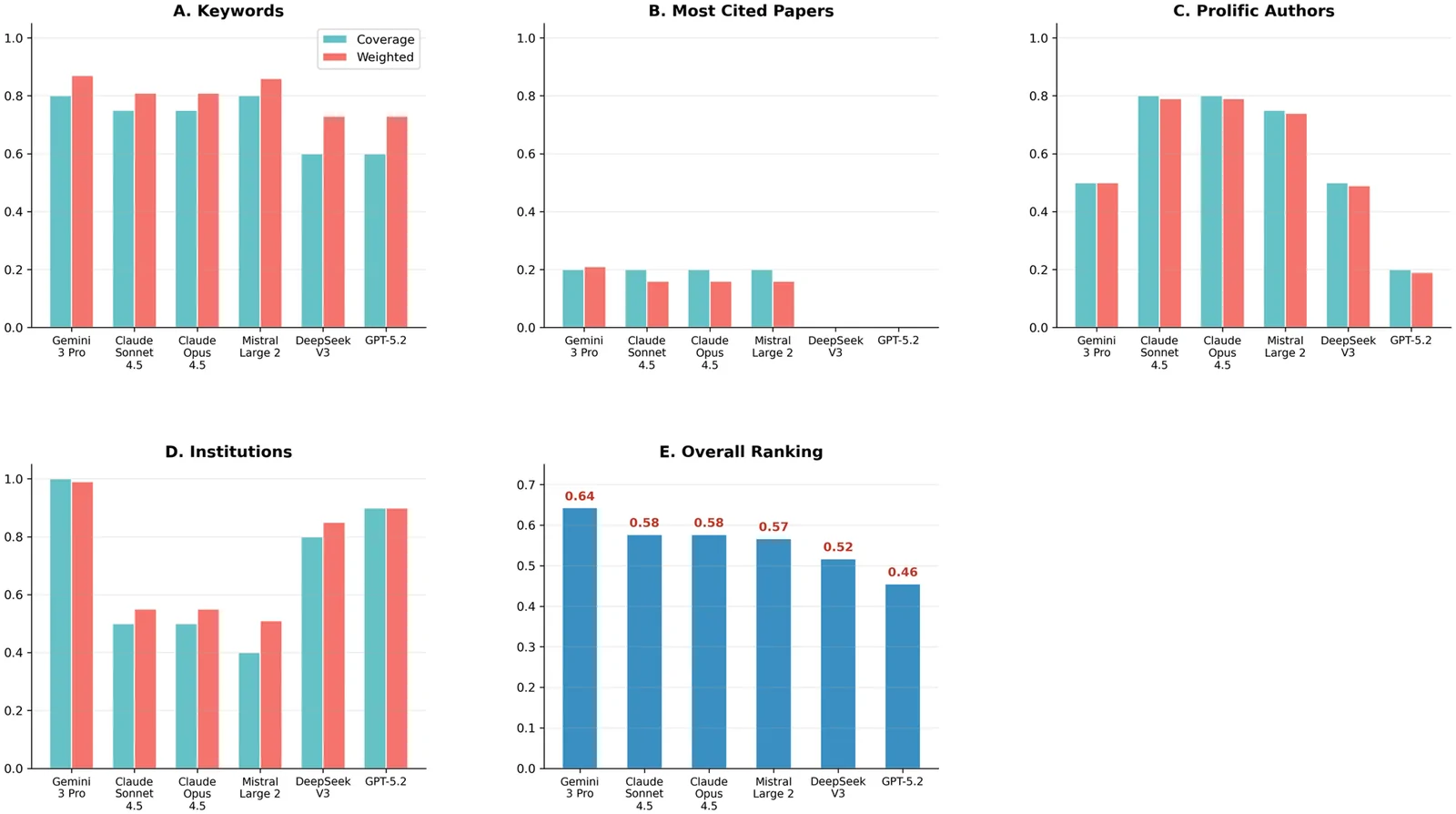
Benchmark Performance of Six Frontier LLMs on UK Biobank Knowledge Retrieval Tasks (January 2026). **(A)** Keywords: Keyword recognition benchmark comparing Coverage Score (proportion of top 20 keywords matched) and Weighted Coverage Score (frequency-weighted) across all six models. Gemini 3 Pro and Mistral Large 2 achieved highest coverage (0.80), followed by Claude models (0.75). GPT-5.2 and DeepSeek V3 scored 0.60. **(B)** Most Cited Papers: All models struggled with this challenging task. Gemini 3 Pro scored highest (Coverage 0.20, Weighted 0.21), while GPT-5.2 and DeepSeek V3 failed to identify any matching papers (0.00). **(C)** Most Prolific Authors: Each model’s output is assessed for matching the top 20 authors by publication count. Coverage Scores capture how many authors each LLM mentions, while Weighted Coverage Scores emphasize authors with more total publications. **(D)** Applicant Institutions: Shows performance in detecting the 10 most frequent UK Biobank applicant institutions. Scores are weighted according to the institution’s application counts. **(E)** Overall Ranking: Overall weighted coverage ranking across all four tasks. Gemini 3 Pro leads (0.643), followed by Claude Sonnet 4.5 and Claude Opus 4.5 (0.577 each), Mistral Large 2 (0.567), DeepSeek V3 (0.517), and GPT-5.2 (0.455).

### Baseline comparisons

To contextualize LLM performance and assess whether observed results reflect meaningful biobank-specific knowledge retrieval rather than random term association, we implemented a random baseline across all four tasks. For each task, we extracted the full set of relevant terms from UK Biobank metadata (keywords from publication abstracts (Schema 19), author names and paper titles, and institutional affiliations from approved applications (Schema 27)). Random baseline responses were then generated by randomly sampling from these term pools, matching the expected output length and formatting to resemble typical LLM completions.

We created 10,000 independent random samples per task to build empirical distributions of baseline performance. These outputs were scored using the same semantic matching and frequency-weighting pipeline applied to the LLM responses, specifically the Weighted Coverage Score metric (Table 1) This metric was chosen for the baseline comparison because it measures factual retrieval performance (the proportion of ground-truth entities retrieved, weighted by their frequency or citation impact) and can be computed identically for both coherent LLM outputs and randomly assembled term lists. The six multidimensional qualitative measures (Semantic Accuracy, Factual Correctness, Domain Knowledge, Reasoning Quality, Response Depth, and Biobank Specificity) were not applied to the random baseline, as several of these dimensions, particularly Reasoning Quality and Response Depth, require coherent, structured text with logical argumentation and thematic synthesis, properties that randomly sampled term lists lack by construction. The resulting Weighted Coverage Score distributions served as null models for statistical testing. Each model’s Weighted Coverage Score was compared against this empirical distribution of 10,000 random-baseline scores using an exact one-sided exceedance test (the proportion of baseline scores reaching or exceeding the model’s score); no baseline sample reached any model’s score, giving p < 10^−^^4^ for all six models.

**Table 1:** Weighted Coverage Scores for Six Frontier LLMs Across Four UK Biobank Benchmark Tasks. Each cell reports the Weighted Coverage Score, which measures the proportion of ground-truth entities retrieved by each model, weighted by entity frequency or citation impact in UK Biobank metadata (Schemas 19 and 27). The Overall score is the mean Weighted Coverage Score across all four tasks.

Model	Keywords	Papers	Authors	Institutions	Overall
Gemini 3 Pro	0.87	0.21	0.50	0.99	0.643
Claude Sonnet 4.5	0.81	0.16	0.79	0.55	0.577
Claude Opus 4.5	0.81	0.16	0.79	0.55	0.577
Mistral Large 2	0.86	0.16	0.74	0.51	0.567
DeepSeek V3	0.73	0.00	0.49	0.85	0.517
GPT-5.2	0.73	0.00	0.19	0.90	0.455

## Results

### Ground truth results

First, we present the analysis of the results that we obtained by parsing both Schema 19 (UK Biobank’s 8,553 publications) and Schema 27 (UK Biobank’s 15,046 research applications). Fig 1 summarizes the UK Biobank publication landscape (panel A) together with the results of our four prompting questions (panels B to E).

### Analysis of top keywords in publications

The most prevalent keyword was “Humans”, appearing 6,547 times, followed by “Female” (3,469), “Male” (3,277) and “Middle Aged” (2,774) (Fig 1B). These results suggest a strong emphasis on human-related studies, with a particular focus on sex and age demographics.

Geographical representation was also notable, with “United Kingdom” ranking among the top five (2,689 occurrences). Key methodological terms such as “Genome-Wide Association Study” (1,940), “Mendelian Randomization Analysis” (751), and “Prospective Studies” (1,304) indicated a research focus on genetic epidemiology and population-based cohort analyses.

Health-related keywords, including “Cardiovascular Diseases” (711) and “Diabetes Mellitus, Type 2” (544), suggest a strong interest in chronic disease genetics. Other notable terms such as “Risk Factors” (2,264), “Genetic Predisposition to Disease” (1,336) and “Multifactorial Inheritance” (508) further highlight the relevance of polygenic risk and complex trait analyses in these studies.

### Most cited articles related to the UK biobank

An analysis of the most cited research articles associated with the UK Biobank (Fig 1C) highlights key areas of scientific interest and impact. The highest-cited publication is on *Comparison of Sociodemographic and Health-Related Characteristics of UK Biobank Participants with Those of the General Population* (published in the *American Journal of Epidemiology*) [13], which has accumulated 2,548 citations (according the UK Biobank’s metadata schema), underscoring the significance of demographic and health-related factors in large-scale biobank studies.

Studies leveraging genome-wide association studies (GWAS) dominate the citation rankings, reflecting the widespread use of the UK Biobank for uncovering genetic risk factors. The second most cited paper, *Genome-wide association analyses identifying 44 risk variants* (*Nature Genetics*) [14], has 2,419 citations, followed closely by research on *Genome-wide polygenic scores for common diseases* (*Nature Genetics*, 2,291 citations) [15].

The impact of body composition on mortality is also a prominent topic, with *Body-mass index and all-cause mortality* (*The Lancet*) accumulating 1,965 citations. Similarly, *Gene discovery and polygenic prediction from a GWAS* (*Nature Genetics*) [16] has received 1,958 citations, reinforcing the role of biobank-scale datasets in predictive genomics.

Mental health research has also gained significant attention, as seen in *Genome-wide meta-analysis of depression* (*Nature Neuroscience*, 1,843 citations) [17], which highlights the genetic basis of psychiatric conditions. Additionally, *Genome-wide meta-analysis identifies new loci and functional pathways influencing Alzheimer’s disease risk* (*Nature Genetics*, 1,777 citations) [18] and *Meta-analysis of GWAS for height* (*Human Molecular Genetics*, 1,710 citations) [19] further illustrate the diversity of genomic research leveraging the UK Biobank.

Beyond genetics, neuroimaging and brain-related studies are also represented, with *Multimodal population brain imaging in the UK Biobank prospective study* (*Nature Neuroscience*) accumulating 1,577 citations [20]. Finally, *Identification of novel risk loci, causal insights, and heritable risk for Parkinson’s disease: a meta-analysis of genome-wide association studies* (*The Lancet Neurology*) has 1,548 citations [21].

### Authorship and institutional contributions to UK biobank research

The analysis of publication output reveals the most prolific researchers contributing to studies leveraging the UK Biobank (Fig 1D). George Davey Smith ranks as the most published author, with 122 publications, followed by Naveed Sattar (119), Kari Stefansson (105), and Caroline Hayward (94). Several other notable researchers, including Stephen Burgess (93), Wei Cheng (92), and Carlos Celis-Morales (79), also feature prominently, indicating strong engagement in biobank-based research from diverse fields such as epidemiology, genomics, and cardiometabolic health.

In terms of institutional engagement (Fig 1E), the University of Oxford leads with 186 applications, reflecting its central role in UK Biobank-related studies. The University of Cambridge (74 applications) and Imperial College London (69 applications) follow, showcasing their strong contributions to biobank-driven investigations. Other major UK institutions, such as University College London (69), University of Edinburgh (62), and University of Manchester (61), demonstrate widespread institutional involvement.

Interestingly, UK Biobank Ltd itself appears as a key applicant with 60 applications, likely reflecting in-house research and collaborative projects. Notably, international representation is observed with Sun Yat-Sen University (39 applications), indicating global interest in UK Biobank data.

### LLM performance across four biobank-relevant tasks

Fig 2 summarizes the performance of all tested Large Language Models (LLMs) in retrieving information about UK Biobank research from our reference corpora. We evaluated four specific tasks (covering keywords in publications, top cited papers, top authors, and top applicant institutions) using both Coverage Score (breadth of matched concepts) and Weighted Coverage Score (concepts weighted by their relative importance or frequency). In the case of a score of 0.0 this means the model was not able to retrieve any of the “ground truth” results. We aggregated each model’s performance across the four tasks to produce an overall benchmark ranking.

### Keyword retrieval

Fig 2A compares each model’s ability to identify the top 20 most frequent keywords in the UK Biobank literature. Gemini 3 Pro and Mistral Large 2 achieved the highest keyword coverage (0.80), each identifying 16 of the top 20 keywords, with weighted scores of 0.87 and 0.86 respectively. The Claude models (Opus 4.5 and Sonnet 4.5) followed closely with coverage of 0.75 (weighted 0.81), identifying 15 keywords. GPT-5.2 and DeepSeek V3 both achieved 0.60 coverage (weighted 0.73), identifying 12 keywords each.

### Top cited papers

Fig 2B displays results for identifying the most highly cited articles based on our curated list. All models struggled with this challenging task. Gemini 3 Pro performed best with a coverage score of 0.20 (weighted 0.21), identifying 2 of the top 10 most cited papers. Claude Opus 4.5, Claude Sonnet 4.5, and Mistral Large 2 all achieved identical coverage of 0.20 (weighted 0.16), also identifying 2 papers each. GPT-5.2 and DeepSeek V3 failed to identify any of the top cited papers (coverage 0.00).

### Most prolific authors

Fig 2C evaluates retrieval of the top 20 UK Biobank authors by publication count. The two Claude models led this task, each reaching 0.80 coverage and 0.79 weighted coverage, and recovered many of the highest-output authors, including George Davey Smith, Naveed Sattar, and Kari Stefansson. Mistral Large 2 followed closely (0.75 coverage, 0.74 weighted). Gemini 3 Pro and DeepSeek V3 each reached about 0.50 coverage (0.50 and 0.49 weighted, respectively), while GPT-5.2 performed weakest, recovering roughly a fifth of the reference authors (0.20 coverage, 0.19 weighted).

### Top applicant institutions

Fig 2D focuses on the top 10 institutions with the highest number of UK Biobank applications. Gemini 3 Pro achieved perfect institutional coverage (1.00), correctly identifying all top 10 applicant institutions. GPT-5.2 followed with 0.90 coverage (weighted 0.90), identifying 9 institutions. DeepSeek V3 achieved 0.80 coverage (weighted 0.85), identifying 8 institutions. The Claude models (Opus 4.5 and Sonnet 4.5) scored 0.50 coverage (weighted 0.55), identifying 5 institutions each. Mistral Large 2 scored lowest on this task with 0.40 coverage (weighted 0.51), identifying 4 institutions.

### Overall accuracy ranking

We computed each model’s average Weighted Coverage across all four tasks to generate an overall ranking (Fig 2E, Table 1). Across the four benchmark tasks, Gemini 3 Pro achieved the highest overall weighted coverage score (0.643), followed by Claude Sonnet 4.5 and Claude Opus 4.5 (both 0.577), Mistral Large 2 (0.567), DeepSeek V3 (0.517), and GPT-5.2 (0.455). The overall coverage scores (unweighted) followed a similar pattern: Gemini 3 Pro (0.625), Claude models (0.563), Mistral Large 2 (0.538), DeepSeek V3 (0.475), and GPT-5.2 (0.425).

### Semantic fidelity and interpretive competence across models

To rigorously assess the interpretive capabilities of large language models (LLMs) in the context of biobank-specific information retrieval, we implemented a multidimensional evaluation framework encompassing six orthogonal performance dimensions: semantic accuracy, reasoning quality, domain knowledge, factual correctness, response depth, and biobank specificity. These dimensions were designed to interrogate not only lexical alignment, but also the structural and inferential characteristics of model outputs.

**Fig 3:**
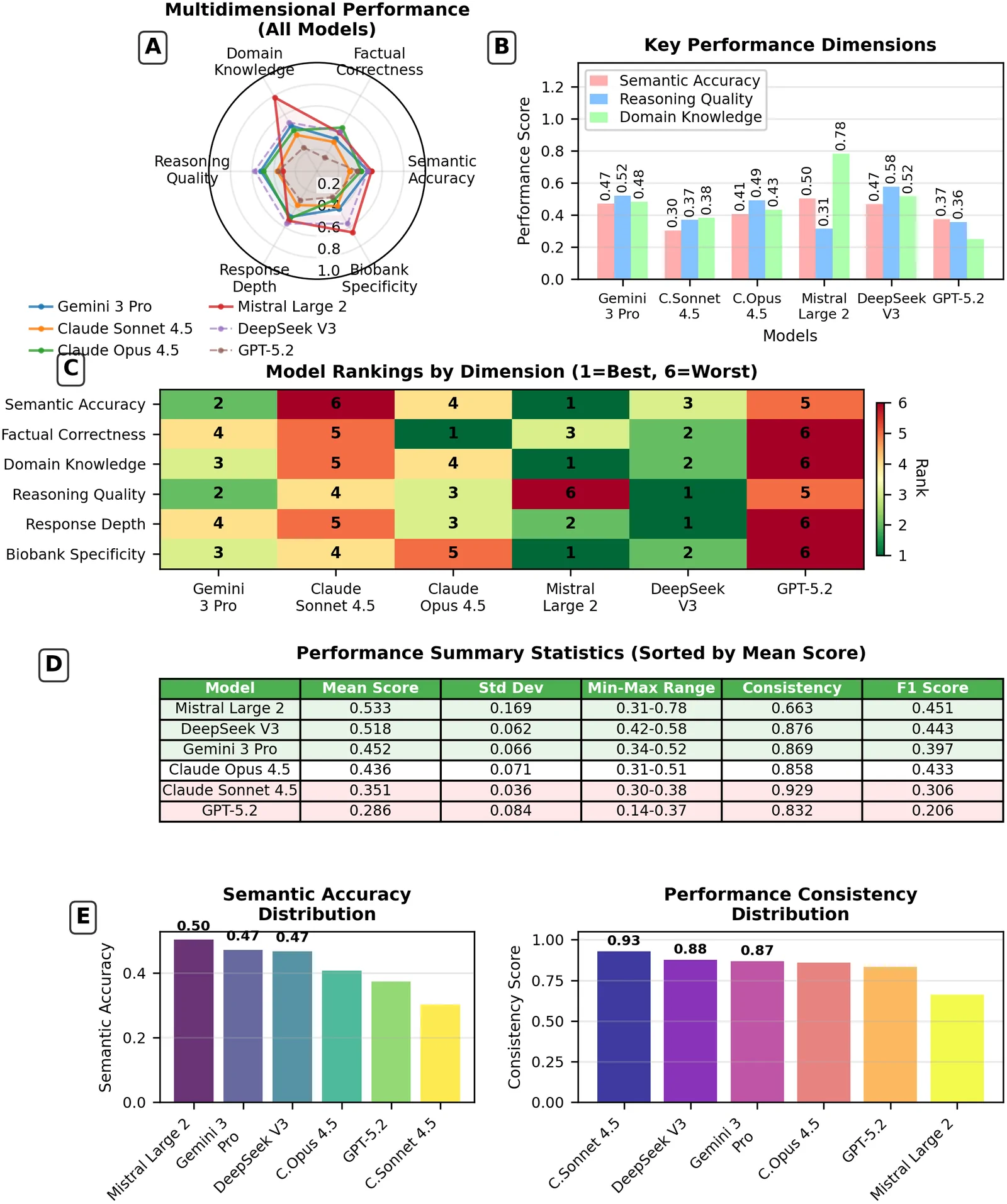
Multidimensional Performance Analysis of Six Frontier Large Language Models on UK Biobank Benchmark (January 2026). **(A)** Radar plot showing normalised scores (0–1 scale) across six evaluation dimensions for all six models. Each axis represents one dimension: Semantic Accuracy, Factual Correctness, Domain Knowledge, Reasoning Quality, Response Depth, and Biobank Specificity. Mistral Large 2 (red) shows the highest overall mean score, while DeepSeek V3 (purple) demonstrates strength in reasoning quality and response depth. **(B)** Grouped bar chart comparing three key evaluation dimensions (Semantic Accuracy, Reasoning Quality, Domain Knowledge) across all six models. Values labelled directly on bars. **(C)** Heatmap of model rankings (1 = best, 6 = worst) for each of the six evaluation dimensions. Colour gradient: green indicates top ranks, red indicates bottom ranks. **(D)** Summary statistics table displaying Mean Score, Standard Deviation, Score Range, Consistency Score, and F1 Score for each model, sorted by Mean Score in descending order. **(E)** Distribution plots showing model-wise performance for Semantic Accuracy (left panel) and Performance Consistency (right panel). Models ranked by respective scores. Claude Sonnet 4.5 shows highest consistency (0.929) across dimensions.

Fig 3A presents a radar chart capturing each model’s relative performance across all six dimensions. Mistral Large 2 demonstrated the most uniformly high scores across multiple dimensions, particularly in domain knowledge and biobank specificity. DeepSeek V3 showed strong and balanced performance across reasoning quality and response depth. Gemini 3 Pro performed strongly across semantic accuracy and reasoning quality, ranking second on both. GPT-5.2 showed more heterogeneous patterns, achieving moderate scores in some dimensions but lower scores in factual correctness and domain knowledge.

Fig 3B isolates three core metrics (semantic accuracy, reasoning quality, and domain knowledge) and presents absolute performance values. Each model showed distinct strengths across dimensions. Mistral Large 2 achieved the highest semantic accuracy (0.50), followed by Gemini 3 Pro (0.47), reflecting strong alignment between model outputs and reference content. DeepSeek V3 led in reasoning quality (0.58), followed by Gemini 3 Pro (0.52), suggesting effective thematic synthesis and interpretive structuring. Mistral Large 2 also demonstrated the strongest domain knowledge (0.78), followed by DeepSeek V3 (0.52), indicating appropriate contextualization of methodological constructs (e.g., Mendelian randomization, longitudinal designs). GPT-5.2 and Mistral Large 2 showed the weakest reasoning quality scores (0.36 and 0.31, respectively), producing more variable outputs across dimensions.

Fig 3C ranks each model across the six metrics. The rank heatmap reveals that Mistral Large 2 was consistently positioned in the top two across most categories, ranking first in semantic accuracy, domain knowledge, and biobank specificity. DeepSeek V3 ranked first in reasoning quality and response depth. Claude Opus 4.5 ranked first in factual correctness.

Aggregate performance statistics are reported in Fig 3D, with models sorted by mean composite score. Mistral Large 2 led with a mean score of 0.533, followed closely by DeepSeek V3 (0.518) and Gemini 3 Pro (0.452). Claude Sonnet 4.5 demonstrated the highest consistency (0.929), denoting low intra-model variance despite a lower overall mean score. DeepSeek V3 yielded the highest consistency among top performers (0.876), reflecting stable performance across task types.

Finally, Fig 3E visualizes the distribution of semantic accuracy and consistency scores. Mistral Large 2 achieved the highest semantic accuracy (0.50), followed by Gemini 3 Pro and DeepSeek V3 (0.47 each), with the remaining models clustering between 0.30 and 0.41. For consistency, Claude Sonnet 4.5 led (0.929), followed by DeepSeek V3 (0.876) and Gemini 3 Pro (0.869). Notably, Claude Sonnet 4.5 exhibited the highest consistency despite a lower overall mean score, suggesting uniformly moderate performance rather than uniformly high accuracy across dimensions.

While most models surpassed baseline lexical recall, only a minority (chiefly Gemini 3 Pro and, to a lesser extent, the Claude models and Mistral Large 2) demonstrated semantically robust, domain-aware, and contextually coherent representations of biobank-derived knowledge.

### Comparison to random baseline performance

**Fig 4:**
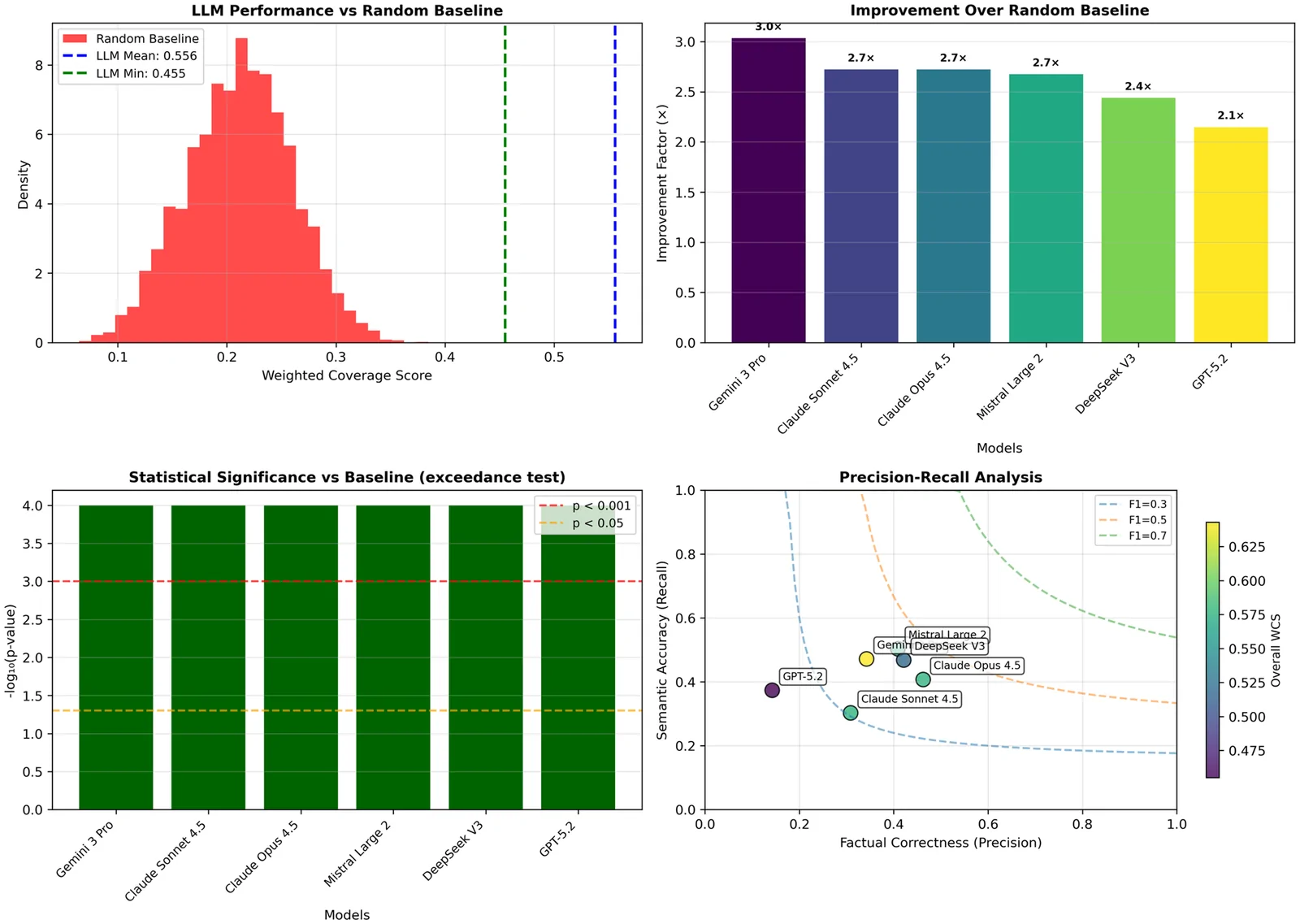
LLM Weighted Coverage Performance Compared to Random Baseline. All panels use the Weighted Coverage Score (mean across all four benchmark tasks: Keywords, Papers, Authors, and Institutions) as the evaluation metric. This is the same “Overall” score reported in Table 1. The Weighted Coverage Score measures the proportion of ground-truth entities retrieved by each model, weighted by entity frequency or citation impact in UK Biobank metadata. Qualitative evaluation dimensions (Reasoning Quality, Response Depth, etc.) are not applied to random outputs, as they require coherent text. **(A)** Density histogram showing the distribution of Weighted Coverage Scores for the random baseline (n = 10,000 iterations of random term sampling from UK Biobank vocabulary pools; baseline mean Weighted Coverage Score 0.212). Vertical dashed lines indicate the mean Weighted Coverage Score across all six LLMs (blue, 0.556) and the minimum LLM score (green, 0.455). All six LLMs fall far outside the random baseline distribution. **(B)** Improvement factors for each LLM over the random baseline, calculated as (Model Weighted Coverage Score) / (Random Baseline Mean Weighted Coverage Score). All models achieve 2× to 3× improvement over chance, indicating genuine encoding of biobank-specific knowledge rather than coincidental term overlap. **(C)** Statistical significance of each LLM versus the random baseline, tested using an exact one-sided exceedance test. The y-axis displays -log10(p-value); horizontal lines indicate significance thresholds at p < 0.001 (red) and p < 0.05 (orange). All models achieve p < 0.001. **(D)** Precision-Recall analysis for entity extraction (authors and institutions) across all models. Point color indicates Overall Weighted Coverage Score (colorbar). Dashed curves represent F1 score iso-lines at 0.3, 0.5, and 0.7. Model names are annotated adjacent to each data point.

To validate that LLM performance reflects genuine biobank knowledge rather than coincidental term overlap, we compared each model’s Weighted Coverage Score (Table 1) against a random baseline distribution (Fig 4). All six models substantially outperformed the random baseline. The mean Weighted Coverage Score across LLMs (0.556) exceeded the random baseline mean (0.212) by 2.6× (Fig 4A and 4B); per-model improvements ranged from 2.1× to 3.0×, all reaching statistical significance at p < 0.001 (exact one-sided exceedance test; Fig 4C). These results confirm that the models encode meaningful biobank-specific knowledge acquired during pre-training.

Fig 4 (top-left panel) displays the kernel density distribution of random baseline scores (red), overlaid with the minimum (0.455) and mean (0.556) performance scores achieved by LLMs. The non-overlapping distributions confirm that even the weakest model (GPT-5.2) significantly outperformed the best-case baseline. Top-right, we show relative improvement factors over baseline performance. Gemini 3 Pro led with a 3.0× improvement, followed by Claude Sonnet 4.5 and Claude Opus 4.5 (2.7× each), and Mistral Large 2 (2.7×). DeepSeek V3 achieved 2.4× improvement, while GPT-5.2, the weakest performer, still exceeded the baseline by 2.1×.

The bottom-left panel displays −log10(p-value) comparisons from the exact one-sided exceedance test between each LLM and the random baseline distribution. Because no random baseline sample (of 10,000) reached the Weighted Coverage Score of any model, all six models separate from the baseline with the same maximal significance (exact one-sided p < 10^−4^); the test does not distinguish an ordering among them. Even GPT-5.2, the weakest performer, separates from the baseline at this same high significance (p < 0.001).

Finally, Fig 4 (bottom-right) introduces a precision-recall analysis contextualized by interpolated F1 isocurves. Gemini 3 Pro attained the best balance of high recall and precision, while DeepSeek V3 and GPT-5.2 exhibited high recall. The Claude models showed balanced precision-recall profiles.

## Discussion

This study benchmarks the ability of general-purpose large language models (LLMs) to recall biobank-relevant information, using curated UK Biobank metadata and literature as ground truth. The analysis draws from 8,553 publication records and over 15,000 application records to evaluate LLM performance across four core tasks: keyword synthesis, citation retrieval, author recognition, and institutional mapping. Our results provide a structured comparison of six frontier models (as of January 2026) using coverage, semantic, and inferential metrics.

### Core themes in UK biobank research

Analysis of the UK Biobank literature highlights two dominant thematic clusters: demographic characterization and genetic epidemiology. The frequent appearance of terms such as “Female,” “Male,” “Middle Aged,” and “United Kingdom” reflects the resource’s focus on population-level stratification across sex, age, and region. These demographic markers are complemented by methodological terms like “Genome-Wide Association Study” and “Mendelian Randomization Analysis,” underscoring the program’s central role in uncovering the genetic architecture of complex diseases. The most cited studies further reflect this pattern, with a strong emphasis on polygenic scoring, causal inference, and risk stratification across cardiometabolic and neurological domains.

Beyond keywords and citations, metadata analyses reveal a robust institutional footprint centered on UK-based institutions, with Oxford, Cambridge, and Imperial College London leading in application volume. Prominent individual researchers (including George Davey Smith, Naveed Sattar, and Kari Stefansson) emerged consistently in both citation and authorship metrics, confirming the consistency and quality of the ground truth corpus.

### Model performance and semantic competence

While all models substantially outperformed a null baseline across tasks, their relative strengths varied sharply depending on the evaluation dimension.

Gemini 3 Pro achieved the highest overall Weighted Coverage Score (Table 1). In the multidimensional evaluation, Mistral Large 2 recorded the highest overall mean score (0.533) and led in semantic accuracy (0.50) and domain knowledge (0.78). Claude Sonnet 4.5 demonstrated the greatest consistency across dimensions (0.929), indicating stable performance despite a lower overall mean. DeepSeek V3 led in reasoning quality (0.58) and response depth (0.56), with Gemini 3 Pro second in reasoning quality (0.52). Both Claude models showed strong author identification capabilities (0.79 weighted coverage on the authors task).

GPT-5.2 showed strong performance on institutional recognition (0.90) but struggled with author identification (0.20). DeepSeek V3 achieved moderate to strong scores across dimensions, with institutional mapping (0.85) being its strongest task. Performance variation across task types highlights the importance of multi-dimensional evaluation.

Importantly, the precision-recall analysis indicates divergent calibration profiles. Gemini 3 Pro balanced sensitivity and specificity effectively. DeepSeek V3 achieved moderate recall (0.47) with moderate precision (0.42), while GPT-5.2, despite high recall, showed diminished precision, indicating a tendency toward overgeneration of partially relevant content. The Claude models and Mistral Large 2 exhibited more conservative recall with moderate to high precision. These findings point to fundamental differences in how LLMs prioritize inference, lexical matching, and factual grounding under open-ended biomedical prompting.

### Random baseline comparison and statistical validation

To rigorously test whether LLM outputs reflect structured knowledge retrieval rather than stochastic lexical overlap, we benchmarked all models against a random baseline across all task categories. Random outputs were constructed by sampling from the UK Biobank term distributions (Schemas 19 and 27), with structural formatting and token count matched to actual model completions. These baselines yielded uniformly poor performance, with Weighted Coverage Scores well below those of every model (baseline mean 0.212) and no overlap with the empirical distribution of LLM-generated scores.

In contrast, all LLMs achieved substantial performance gains relative to this null distribution. Gemini 3 Pro achieved the largest improvement (exceeding the baseline by a factor of 3.0×) followed by Claude Sonnet 4.5 and Claude Opus 4.5 (2.7× each) and Mistral Large 2 (2.7×). Even the weakest model, GPT-5.2, surpassed the baseline by 2.1×. These results suggest that model outputs cannot be attributed to frequency priors or random recombination of biomedical terms.

Statistical validation using exact one-sided exceedance tests confirmed significant separation between LLM and baseline responses for all models, with p-values < 0.001. This robust separation reinforces that LLM outputs reflect non-random retrieval of biobank-relevant information rather than chance lexical associations.

### Interpretation and practical implications

The results of this benchmarking study highlight the emerging utility of general-purpose LLMs as viable tools for semantic querying and interpretive synthesis within large-scale biomedical corpora such as the UK Biobank. These capabilities position top-tier LLMs as promising candidates for tasks such as metadata harmonization, cohort stratification, and hypothesis generation within translational research pipelines.

At the same time, the analysis reveals considerable variance across models and tasks. Certain systems exhibited strengths in reasoning or domain-specific vocabulary yet lacked precision or factual grounding. Others demonstrated high recall but poor calibration, generating outputs that were partially relevant but prone to overgeneralization. These discrepancies emphasize the limitations of traditional evaluation metrics and the need for more rigorous, task-specific assessment frameworks in biomedical NLP.

Crucially, the observed variation in performance consistency underscores the importance of reproducible and interpretable benchmarking practices. Without fine-grained evaluation protocols, differences in LLM outputs may be masked or misinterpreted, particularly in high-stakes settings where domain specificity and inferential accuracy are critical.

### Benchmark value and reusability

A natural question concerns the lasting value of a benchmark in the rapidly evolving LLM landscape, where model rankings may shift with each new release. We argue that our contribution extends beyond the specific rankings reported here in three important ways. First, we provide a reusable methodology and open-source framework for evaluating LLMs on biomedical research tasks. The code, ground truth datasets, and evaluation pipeline (available at https://github.com/manuelcorpas/LLM_4_UKB) can be readily applied to benchmark future models, enabling longitudinal tracking of progress in this domain. Second, we establish baseline performance expectations for this task category. Our random baseline analysis demonstrates that current LLMs genuinely encode biobank knowledge (achieving 2× to 3× performance above chance), providing a calibration point against which future models and methodologies can be compared. Third, the multidimensional evaluation framework itself represents a methodological contribution. The six dimensions we define (semantic accuracy, factual correctness, domain knowledge, reasoning quality, response depth, and biobank specificity) can be adapted to evaluate LLM performance on other specialized biomedical knowledge retrieval tasks beyond the UK Biobank context. Finally, the specific findings about model characteristics provide actionable insights for researchers selecting tools for biobank-related tasks today. For example, Gemini 3 Pro’s strength in institutional knowledge and keyword recognition, the Claude models’ superior author identification capabilities, and the general challenge of biobank specificity observed across all models inform practical tool selection. The framework ensures these insights can be systematically updated as new models emerge.

### Limitations

While our evaluation framework captures a broad spectrum of interpretive capabilities, several limitations should be acknowledged. First, the study focuses on retrospective task performance using structured prompts and predefined ground-truth corpora; it does not evaluate models in generative or forward-looking tasks such as hypothesis development, data harmonization, or longitudinal pattern inference. These use cases are critical to translational research and warrant dedicated methodological assessment.

Second, although our semantic scoring pipeline incorporates embedding-based similarity and expert review, it remains constrained by the representation biases of the underlying language model used for vectorization. Semantically equivalent responses that diverge lexically may be underweighted if they fall below the cosine similarity threshold. Future approaches could incorporate multi-embedding ensembles or dynamic thresholding to improve sensitivity.

Third, our study benchmarks general-purpose models accessed via public prompts. We did not include domain-specialized models fine-tuned on biomedical literature (e.g., PubMedBERT [22] or Galactica [23]) or retrieval-augmented generation (RAG) architectures, which may offer enhanced factual grounding and context integration. Including such systems in future comparisons could provide valuable insight into the tradeoffs between generalizability and specialization.

Finally, while our random baseline offers a stringent null model for statistical benchmarking, comparisons against established symbolic or rule-based information retrieval (IR) systems were not performed. Including classical baselines such as BM25 [24], concept graph traversal [25], or MeSH-based indexing could enrich future evaluations and help disentangle the contributions of semantic modeling versus domain priors [26].

### Future directions

As LLMs become increasingly embedded within biomedical research and clinical informatics pipelines, future evaluation paradigms must evolve to capture their full operational scope. Beyond entity retrieval and semantic matching, there is a pressing need to benchmark models on complex reasoning tasks, including multi-hop inference, causal explanation, and counterfactual generation [27]. Incorporating interpretability audits (such as attribution mapping and calibration diagnostics [28]) will also be critical for building trust and ensuring model transparency in high-stakes settings.

Further, expanding evaluation to clinically actionable endpoints (such as treatment prioritization, risk score generation, or eligibility screening) would better align benchmarking efforts with translational objectives [29]. These tasks require not only semantic alignment but robust integration of structured medical logic, patient heterogeneity, and evolving clinical guidelines [30].

Finally, the incorporation of temporally-aware prompts, multimodal inputs (e.g., tabular biomarkers, imaging reports), and structured biomedical ontologies (e.g., SNOMED CT [31], UMLS, or MeSH) represents a promising frontier for enhancing factual grounding and context sensitivity [32]. Benchmarking models in these contexts will be essential for determining their readiness to support real-world biomedical decision-making, from cohort design to individualized care planning.

The LLM landscape continues to evolve rapidly. The models evaluated here: Gemini 3 Pro, Claude Opus 4.5, Claude Sonnet 4.5, GPT-5.2, Mistral Large 2, and DeepSeek V3, represent the frontier as of January 2026. This evaluation updates our initial experiments conducted in late 2024 [33], which included earlier model generations (Gemini 2.0 Flash, Claude Sonnet 3.5, GPT-4o, among others). The transition from our 2024–2026 evaluation demonstrates both the reusability of our benchmark framework and the continued advancement in model capabilities. We encourage future studies to apply our open-source framework to evaluate subsequent model releases and contribute to longitudinal tracking of progress in biobank-related knowledge retrieval.

## Conclusion

This study introduces a multidimensional benchmarking framework for evaluating large language models (LLMs) in the context of the UK Biobank. By leveraging structured prompts and curated outputs, we demonstrate that general-purpose LLMs vary widely in their ability to retrieve, contextualize, and synthesize domain-specific knowledge.

Our results reveal that top-performing models (particularly Gemini 3 Pro) consistently exhibit high semantic accuracy and stable performance across diverse query types, whereas others display fragmented or error-prone behavior. Crucially, we show that coverage-based metrics alone are insufficient to capture these differences, underscoring the importance of deeper, task-sensitive evaluation criteria for biomedical applications.

Beyond benchmarking, our analysis also surfaces key thematic insights. Research involving UK Biobank data is dominated by demographic stratification (e.g., sex and age), genetic epidemiology (notably GWAS and Mendelian randomization), and chronic disease phenotypes such as cardiovascular disease and type 2 diabetes. We also identify leading contributors, including prolific authors and high-application institutions, that shape the research landscape. These findings reinforce the centrality of the UK Biobank in population-scale genomics and highlight emerging areas of opportunity for more diverse or underexplored domains.

As LLMs become increasingly embedded in biomedical research workflows, the ability to systematically characterize both their interpretive capabilities and the structures of the datasets they interrogate will be essential. The framework and findings presented here offer a rigorous foundation for future model development and evaluation, and also a lens into one of the world’s most important biomedical data resources.

## Supporting information

S1 Text Reproducibility of Coverage Score and Weighted Coverage Score. Detailed explanation of the methodology for calculating Coverage Score and Weighted Coverage Score metrics, including semantic similarity matching using sentence embeddings, synonym normalization, and frequency weighting approaches.
(DOCX)

S1 Table Scoring Rubrics for Multidimensional Evaluation Framework. Detailed scoring rubrics for each of the six evaluation dimensions (Semantic Accuracy, Factual Correctness, Domain Knowledge, Reasoning Quality, Response Depth, and Biobank Specificity) used in the multidimensional evaluation framework, including score ranges and specific criteria for each dimension.
(DOCX)
