# Supplementary material for: Benchmarking large language models for extracting biobank-derived insights into health and disease
Source: PLoS Comput Biol. 2026 Apr 20;22(4):e1014224. doi: 10.1371/journal.pcbi.1014224 (PMC13541248; doi:10.1371/journal.pcbi.1014224)
Supplement: Supplementary file 1 [file pcbi.1014224.s001.docx]

# S1 Text. Reproducibility of Coverage Score and Weighted Coverage Score

To ensure the reproducibility of our evaluation methodology, we provide a detailed explanation of how the Coverage Score and Weighted Coverage Score were calculated in assessing the ability of Large Language Models (LLMs) to retrieve relevant biobank-related information. These metrics quantify how well an LLM’s response captures key biomedical concepts from a predefined reference dataset using a structured approach based on semantic similarity matching.

The Coverage Score measures the proportion of predefined biobank-related terms that appeared in an LLM’s generated response. The reference dataset consisted of frequently used biobank-related terms, compiled from scientific literature. Since biobank research terminology often includes synonyms and alternative phrasings (e.g., “Genome-Wide Association Study” and “GWAS”), we incorporated a synonym-matching approach to account for variations in terminology.

To compute the Coverage Score, LLM-generated responses were first segmented into sentence-level chunks. Each chunk was converted into numerical embeddings using a Sentence Transformer model (all-MiniLM-L6-v2), which maps text into a high-dimensional vector space. Similarly, the reference keywords and their synonyms were also encoded using the same model. Cosine similarity was then computed between each keyword’s embedding and the embeddings of the LLM response segments. If a keyword or any of its synonyms achieved a cosine similarity score of at least 0.20, it was considered a match. The Coverage Score was then computed as:

$$Coverage Score=\frac{Number of Matched Keywords}{Total Number of Reference Keywords}$$

Where:

**Matched Keywords** = Number of predefined reference keywords (or their synonyms) detected in the LLM-generated response.

**Total Reference Keywords** = Total number of predefined reference keywords in the dataset.

Since not all keywords are equally important in biobank-related research, we refined this metric by introducing the Weighted Coverage Score, which assigns greater weight to frequently occurring, high-impact terms. Each keyword was assigned a frequency weight, reflecting its prevalence in biobank-related literature. Instead of treating all matched keywords equally, the Weighted Coverage Score aggregates the frequencies of the detected keywords and normalizes them against the total sum of keyword frequencies. This ensures that widely studied concepts, such as “Genome-Wide Association Study” or “Mendelian Randomization,” contribute more significantly to the final score than less frequently mentioned terms.

Mathematically, the Weighted Coverage Score is defined as:

$$Weighted Coverage Score =\frac{\sum Frequencies of Matched Keywords}{\sum Frequencies of All Reference Keywords}$$

For example, suppose an LLM response contained 10 out of 20 reference keywords. The basic Coverage Score would be:

$$\frac{10}{20}=0.50$$

However, not all keywords contribute equally to biobank-related literature. If the matched keywords collectively appeared 25,000 times in relevant literature, and the total occurrences of all reference keywords summed to 50,000, then the Weighted Coverage Score would be:

$$\frac{25,000}{50,000}=0.50$$

This weighting system refines the Coverage Score by prioritizing high-frequency, high-impact terms, ensuring that concepts central to biobank research contribute more significantly to model evaluation. In this case, even though the basic Coverage Score and Weighted Coverage Score are numerically identical (0.50), the Weighted Coverage Score more accurately reflects the LLM’s ability to retrieve core biobank-related concepts.

A model could, for example, achieve a high Coverage Score by retrieving many low-frequency terms while missing crucial terms such as Genome-Wide Association Study (GWAS) or Mendelian Randomization, which are fundamental to biobank research. The Weighted Coverage Score prevents such distortions, ensuring that the most influential terms receive higher weight in the evaluation.

By incorporating semantic similarity matching, frequency weighting, and synonym normalization, this metric provides a more precise measure of an LLM’s effectiveness in biomedical knowledge retrieval.
