# Supplementary material for: Benchmarking large language models for extracting biobank-derived insights into health and disease
Source: PLoS Comput Biol. 2026 Apr 20;22(4):e1014224. doi: 10.1371/journal.pcbi.1014224 (PMC13541248; doi:10.1371/journal.pcbi.1014224)
Supplement: Supplementary file 2 [file pcbi.1014224.s002.docx]

# S1 Table. Scoring Rubrics for Multidimensional Evaluation Framework

This table provides detailed scoring rubrics for each of the six evaluation dimensions used in the multidimensional evaluation framework.

| **Dimension** | **Score Range** | **Scoring Criteria** |
| --- | --- | --- |
| Semantic Accuracy | 0.0-1.0 | Measured via sentence embedding similarity (all-MiniLM-L6-v2 model) between LLM response and ground truth. Cosine similarity ≥0.75 = High (score 0.8-1.0); 0.50-0.74 = Medium (score 0.5-0.79); <0.50 = Low (score 0.0-0.49). |
| Factual Correctness | 0.0-1.0 | Proportion of named entities (authors, institutions, keywords) in the LLM response that match ground truth entries. Calculated as Precision = TP/(TP+FP), where TP = true positives and FP = false positives. |
| Domain Knowledge | 0.0-1.0 | Assessed by presence of UK Biobank-specific terminology (e.g., “Data-Field,” “Schema,” “UDI,” “Application ID,” “phenotype,” “GWAS”). Score = (matched domain terms in response)/(total domain terms in ground truth). |
| Reasoning Quality | 0.0-1.0 | Evaluated based on logical coherence and justification. Scored via three subscales: (a) presence of causal connectors and logical flow, (b) evidence citation or source attribution, (c) appropriate qualification of uncertainty. Three subscales averaged for final score. |
| Response Depth | 0.0-1.0 | Ratio of information coverage: (unique factual elements in response)/(unique factual elements in comprehensive ground truth). Responses below minimum threshold (50% of expected content) receive penalty; verbosity beyond 2× expected length also penalized. |
| Biobank Specificity | 0.0-1.0 | Proportion of response content specifically addressing UK Biobank context vs. generic biomedical statements. Each sentence classified as biobank-specific or generic; final score = (biobank-specific sentences)/(total sentences). |
