# Supplementary material for: Benchmarking large language models for extracting biobank-derived insights into health and disease
Source: PLoS Comput Biol. 2026 Apr 20;22(4):e1014224. doi: 10.1371/journal.pcbi.1014224 (PMC13541248; doi:10.1371/journal.pcbi.1014224)
Supplement: Supplementary file 5 [file pcbi.1014224.s005.docx]

# Response to Reviewers - Revision 3

**Manuscript ID:** PCOMPBIOL-D-25-00577R2

**Title:** Benchmarking Large Language Models for Extracting Biobank-Derived Insights into Health and Disease

## Reviewer #1

We thank Reviewer #1 for confirming that all previous concerns have been fully resolved.

**R1 Comment:** I appreciate the efforts the authors have put into this revision. My previous comments have been fully resolved. I am satisfied with the current manuscript and recommend acceptance. Congrats!

**Response:** We thank the reviewer for their positive assessment and continued engagement throughout the review process.

## Reviewer #3

We thank Reviewer #3 for the careful re-reading and for drawing our attention to the fact that their original Review Point 3 was not fully addressed in our previous response. We now address both remaining concerns below.

### Point 1: Figure 4 metric definition and rationale for random baseline evaluation

**R3 Comment:** “Some figures and descriptions in the results section lack clarity and should be better linked to the methods. For example, Figure 4 does not specify which metric or result is being plotted (is it coverage, weighted coverage, any multidimensional evaluation, or a composite score? Is it about one of the 4 prompt questions, or an aggregation of all?). The text mentions that random baselines were scored using ‘the same semantic and coverage pipeline,’ but it is unclear whether this includes the multidimensional qualitative measures, which may not be meaningful for random outputs…”

Specifically, for Figure 4, it remains unclear what exactly the measure of “performance” or “improvement” is being evaluated (given that many benchmark measures are discussed but only one measure is plotted and a reader would not know what that is), even with the updated caption. Additionally, the manuscript should clarify the rational of using that particular measure of choice (if one exists), especially what does it mean to evaluate it on the null random outputs (e.g. it may not even make sense to evaluate Reasoning Quality for random outputs).

**Response:** We thank the reviewer for this clarification and apologise for the incomplete response in our previous revision. We now provide a direct and explicit answer.

**What is plotted in Figure 4:** The metric used across all four panels of Figure 4 is the **Weighted Coverage Score**, averaged across all four benchmark tasks (Keywords, Papers, Authors, Institutions). This is the same “Overall” score reported in Table 1 (ranging from 0.455 for GPT-5.2 to 0.643 for Gemini 3 Pro). Weighted Coverage Score measures the proportion of ground-truth entities (keywords, paper titles, author names, institution names) successfully retrieved by each model, weighted by the frequency or citation impact of each entity in the UK Biobank metadata. It is a single, quantitative, automatically computed metric.

**Why this metric was chosen for baseline comparison:** Weighted Coverage Score was selected for the random baseline analysis because it is the primary quantitative metric in our evaluation framework and, critically, it can be computed identically for both LLM outputs and randomly generated outputs. The random baseline was constructed by sampling terms from the same vocabulary pools used to define ground truth (biomedical keywords from Schema 19, author names and institutional affiliations from Schema 27), formatted to match typical LLM response length and structure. Because both LLM and random outputs are scored against the same ground-truth reference lists using the same semantic matching pipeline, the comparison is direct and methodologically consistent.

**Why qualitative dimensions are excluded from the baseline:** The six multidimensional evaluation dimensions described in the Methods (Semantic Accuracy, Factual Correctness, Domain Knowledge, Reasoning Quality, Response Depth, and Biobank Specificity) are designed to assess properties of coherent, structured text. Several of these dimensions, particularly Reasoning Quality and Response Depth, require the presence of logical argumentation, causal connectors, and thematic synthesis. Randomly assembled term lists lack these properties by construction, so applying these qualitative measures to random outputs would not produce meaningful scores. For this reason, the baseline comparison in Figure 4 is restricted to the Weighted Coverage Score, which measures factual retrieval performance and can be evaluated equivalently for both coherent and random outputs. The multidimensional analysis (Figure 3) is applied exclusively to LLM outputs, where it provides additional insight into the qualitative characteristics of each model’s responses.

**Changes made to the manuscript:**

1. **Figure 4 caption** has been rewritten to explicitly identify the metric as “Weighted Coverage Score (mean across all four benchmark tasks)” in each panel description.
2. **Baseline Comparisons section** (Methods) now includes an explicit statement that the random baseline analysis uses only the Weighted Coverage Score, with a justification for why the multidimensional qualitative measures are not applied to random outputs.
3. **Results text** referencing Figure 4 now explicitly names the metric when discussing baseline comparisons.

### Point 2: Figure quality

**R3 Comment:** The other concerns that I mentioned have been sufficiently addressed, but figures are generally not in publication quality. For example, texts on figure 1 are hard to see.

**Response:** We have regenerated all figures with improved readability:

1. **Figure 1:** Regenerated with increased font sizes throughout. Tick labels increased from 5-6pt to 8pt minimum. Panel titles increased from 9pt to 11pt. Axis labels increased from 8pt to 10pt. Paper title labels in Panel C increased from 5pt to 7pt. Institution labels in Panel E given more spacing. The figure has been re-exported as 300 DPI TIFF meeting PLOS specifications (RGB, max 2250x2625 px).
2. **Figures 2-4:** Reviewed for text legibility. Figure 4 axis labels and annotations verified at minimum 8pt for print readability.
3. All figures have been processed through the PLOS NAAS tool to confirm they meet technical requirements.
