# Supplementary material for: Benchmarking large language models for extracting biobank-derived insights into health and disease
Source: PLoS Comput Biol. 2026 Apr 20;22(4):e1014224. doi: 10.1371/journal.pcbi.1014224 (PMC13541248; doi:10.1371/journal.pcbi.1014224)
Supplement: Supplementary file 6 [file pcbi.1014224.s003.docx]

**Response to Reviewers**

Dear Editors,

We are grateful for the constructive feedback provided by the reviewers. Below we provide a point-by-point response to each comment, indicating the changes we have made in the revised manuscript.

**Reviewer #1**

**Comment 1:**

*“The study appears rather straightforward, primarily testing general-purpose LLMs without additional fine-tuning or adaptation to biobank-specific tasks. This limits the novel contributions significantly, as such general capabilities are relatively well-known.”*

**Response:**
We acknowledge this limitation and now clarify in the Introduction and Methods that our goal is to evaluate how general-purpose LLMs perform in realistic, out-of-the-box use cases commonly faced by biomedical researchers. This evaluation, although not involving fine-tuning, reflects how LLMs are actually deployed in many real-world settings without domain-specific adaptation. We now explicitly frame this benchmarking as an important baseline for assessing general model utility in biomedical contexts (see revised Introduction).

**Comment 2:**

*“The evaluation relies heavily on keyword coverage metrics. Such metrics do not sufficiently address the depth, accuracy, or semantic relevance of the retrieved information.”*

**Response:**
We have addressed this by introducing a multidimensional evaluation framework comprising six axes: coverage, semantic accuracy, factual correctness, reasoning quality, domain knowledge, and biobank specificity. This framework provides a more comprehensive and nuanced assessment of model performance (Methods sections “Multidimensional Evaluation of Model Capabilities”, revised pages 4-6; Results section “Semantic Fidelity and Interpretive Competence Across Models”, pages 13–17). These additions move beyond lexical overlap to evaluate semantic and interpretive capabilities.

**Comment 3:**

*“There is no baseline comparison against simpler random-selection methods. The authors acknowledged this limitation but did not sufficiently justify why such baseline tests were omitted.”*

**Response:**
We now include a robust random baseline comparison. We constructed randomized response sets matched to the structure and token count of actual LLM outputs, and scored these using the same pipeline as LLM outputs. All models outperformed the baseline with high statistical significance (p < 0.001), and results are reported in a new Results section and visualized in **Figure 4** (pages 16-17).

**Comment 4:**

*“The study tests a limited set of queries... Including a broader variety of queries, such as those requiring deeper inference, hypothesis generation, or interpretation, would better demonstrate the capabilities or limitations of the models.”*

**Response:**
While we retained the original four query types to ensure reproducibility and comparability, we have expanded our evaluation metrics to specifically capture deeper reasoning and interpretation. This includes new scores for reasoning quality, response depth, and semantic accuracy, which collectively assess how well models infer and synthesise information beyond surface-level retrieval. We also now discuss the need for future inclusion of hypothesis-generation tasks in the Future Directions section (page 20-21).

**Comment 5:**

*“Claims regarding the transformative potential and future routine use of LLMs appear somewhat overstated...”*

**Response:**
We have revised the Abstract, Conclusion, and Discussion sections to tone down speculative claims. We now clearly acknowledge the limitations of current models, the variability in performance, and the need for domain-specific tuning before LLMs can be routinely used for clinical or high-stakes applications (Discussion, page 20).

**Reviewer #2**

**Comment 1:**

*“These four questions are arguably not the best use cases for LLMs…”*

**Response:**
We appreciate this insight. While we kept the original four questions for comparability, we now evaluate model responses using a detailed six-dimensional framework, including reasoning quality and biobank specificity. These metrics help to assess the interpretive and generative aspects of LLMs, even for seemingly straightforward tasks. This reframing strengthens the evaluation without requiring entirely new query types (see Methods and Results).

**Comment 2:**

*“For the subject extraction task… evaluating LLMs based on extracted keywords is not informative.”*

**Response:**
To improve this, we implemented embedding-based semantic similarity matching with a refined threshold and introduced weighting by term frequency. Moreover, we now incorporate manual review of ambiguous matches and evaluate contextual use through semantic accuracy and biobank specificity metrics.

**Comment 3:**

*“It would be more meaningful to evaluate semantic categories or LLM-generated summaries using manual evaluation on selected samples.”*

**Response:**
While a full manual annotation study was beyond scope, we now perform expert-informed manual review for semantic accuracy scoring and ambiguity resolution in embedding matches. This human-in-the-loop approach strengthens the interpretive validity of our evaluation.

We believe these revisions substantially strengthen the manuscript and address the reviewers’ comments in full. Thank you again for your valuable feedback, which has helped improve the rigor and clarity of our work.

Sincerely,

Manuel Corpas and Alfredo Iacoangeli
