# Supplementary material for: Benchmarking large language models for extracting biobank-derived insights into health and disease
Source: PLoS Comput Biol. 2026 Apr 20;22(4):e1014224. doi: 10.1371/journal.pcbi.1014224 (PMC13541248; doi:10.1371/journal.pcbi.1014224)
Supplement: Supplementary file 7 [file pcbi.1014224.s004.docx]

# Response to Reviewers

*Revision 2*

**Manuscript ID:** PCOMPBIOL-D-25-00577R1

**Title:** Benchmarking Large Language Models for Extracting Biobank-Derived Insights into Health and Disease

## Reviewer #1

We thank the reviewer for their positive assessment and the suggestion regarding Gemini 2.5.

**R1 Comment:** *The authors have addressed my concerns adequately. As a minor optional suggestion, would be nice if authors could also benchmark Gemini 2.5, if possible.*

**Response:** We thank the reviewer for this suggestion. We have gone beyond the suggestion and updated our entire benchmark to include the latest frontier models available as of January 2026. Specifically, we now evaluate:

**Gemini 3 Pro** (replacing Gemini 2.0 Flash): Google's latest flagship model

**Claude Opus 4.5** and **Claude Sonnet 4** (replacing Claude 3.5 Sonnet): Anthropic's current generation

**GPT-5.2** (replacing GPT-4o and o1 variants): OpenAI's latest model

**Mistral Large** (updated): Mistral AI's current flagship

**DeepSeek V3** (replacing DeepThink R1): DeepSeek's latest release

This comprehensive update demonstrates the reusability of our benchmark framework while providing timely results with current state-of-the-art models. Notably, Gemini 3 Pro achieves the highest overall score (0.643), consistent with the strong performance of its predecessor in our original evaluation. The full results are presented in updated Table 1 and Figures 3-4.

## Reviewer #3

We thank the reviewer for their thoughtful and constructive feedback. Below we address each point in detail.

### Point 1: Rationale for Benchmark Questions

**R3 Comment:** *The rationale for selecting these four benchmark questions needs to be explicitly stated. While I can infer a logic from the manuscript, readers would benefit from a clear statement upfront about why these questions were chosen for this benchmark.*

**Response:** We have added a dedicated paragraph in the Methods section (Section: Benchmark Question Design) that explicitly articulates the rationale for our four benchmark questions. The text now explains that:

**1. Keyword Synthesis (Q1)** tests the model's ability to aggregate and summarize thematic content across a large corpus, a fundamental task in literature review and trend identification.

**2. Citation-Aware Topic Retrieval (Q2)** requires integrating bibliometric signals (citation counts) with content understanding, reflecting how researchers prioritize impactful literature.

**3. Author Identification (Q3)** evaluates entity recognition and ranking capabilities for person names, essential for identifying collaboration opportunities and key contributors to a field.

**4. Institutional Recognition (Q4)** assesses the model's ability to extract and rank organizational entities from application metadata, critical for understanding the research landscape.

These questions span keyword analysis, bibliometric integration, person entity extraction, and organizational entity extraction: four distinct competencies that collectively represent the range of information needs in biobank research discovery.

### Point 2: Scoring Rubrics for Evaluation Dimensions

**R3 Comment:** *It is unclear how the authors operationalized the multidimensional evaluation framework. The paper would benefit from detailed scoring rubrics or procedures for each dimension.*

**Response:** We have added a new Table (Table S1 in Supporting Information) that provides detailed scoring rubrics for each of the six evaluation dimensions. Additionally, we have added explanatory text in the Methods section referencing these rubrics. The rubrics specify score ranges, criteria, and calculation methods for Semantic Accuracy, Factual Correctness, Domain Knowledge, Reasoning Quality, Response Depth, and Biobank Specificity.

### Point 3: Figure Clarity

**R3 Comment:** *Figure 3 and Figure 4 are unclear and need better captions. It's difficult to understand what's actually being plotted.*

**Response:** We have substantially revised the figure captions to provide explicit detail about what each panel displays. The updated captions now include panel-by-panel descriptions (A, B, C, D, E) specifying the metrics plotted, axis labels, score interpretation, and model comparisons. Figure 3 caption now explains the radar plot dimensions, grouped bar chart metrics, ranking heatmap methodology, summary statistics table contents, and distribution plots. Figure 4 caption clarifies the random baseline distribution, improvement factors calculation, statistical significance testing, and precision-recall analysis methodology.

### Point 4: Benchmark Lasting Value

**R3 Comment:** *The value proposition of the benchmark is not entirely clear. If the model rankings change with new releases, what makes this benchmark worth developing?*

**Response:** We appreciate this important question about the enduring value of our contribution. We have added a paragraph in the Discussion section addressing this directly, explaining that our contribution extends beyond specific rankings in three ways:

**First**, we provide a reusable methodology and open-source framework (available at https://github.com/manuelcorpas/LLM_4_UKB) that can benchmark future models.

**Second**, we establish baseline performance expectations, demonstrating that LLMs genuinely encode biobank knowledge (16-25x above random chance).

**Third**, the six-dimensional evaluation framework itself is a methodological contribution adaptable to other biomedical domains.

Indeed, this revision itself demonstrates the framework's reusability: we have updated from our 2024 models to January 2026 frontier models using the same evaluation pipeline.

## Summary of Changes

| **Section** | **Change** |
| --- | --- |
| Abstract | Updated model names (6 frontier models as of January 2026) |
| Methods | Updated model list; Added Benchmark Question Design rationale; Added reference to Table S2 |
| Results | Updated all results with January 2026 benchmark scores; New Table 1 |
| Supporting Information | Added Table S1 with detailed scoring rubrics |
| Figure 3 | Replaced with updated version; Caption rewritten with panel descriptions |
| Figure 4 | Replaced with updated version; Caption rewritten with panel descriptions |
| Discussion | Added Benchmark Value paragraph; Added Model Evolution Note |

## Updated Results Summary

**Table 1. Overall Performance of Six Frontier LLMs on UK Biobank Benchmark (January 2026)**

| **Rank** | **Model** | **Keywords** | **Papers** | **Authors** | **Institutions** | **Overall** |
| --- | --- | --- | --- | --- | --- | --- |
| 1 | Gemini 3 Pro | 0.87 | 0.21 | 0.50 | 1.00 | **0.643** |
| 2 | Claude Sonnet 4 | 0.81 | 0.16 | 0.79 | 0.55 | **0.577** |
| 3 | Claude Opus 4.5 | 0.81 | 0.16 | 0.79 | 0.55 | **0.577** |
| 4 | Mistral Large | 0.86 | 0.16 | 0.74 | 0.51 | **0.567** |
| 5 | DeepSeek V3 | 0.73 | 0.00 | 0.49 | 0.85 | **0.517** |
| 6 | GPT-5.2 | 0.73 | 0.00 | 0.19 | 0.90 | **0.455** |

*Scores represent weighted coverage (0-1 scale). Overall score is the mean across all four tasks.*

*Revision submitted: January 2026*
