# Supplementary material for: Benchmarking large language models for extracting biobank-derived insights into health and disease
Source: PLoS Comput Biol. 2026 Apr 20;22(4):e1014224. doi: 10.1371/journal.pcbi.1014224 (PMC13541248; doi:10.1371/journal.pcbi.1014224)
Supplement: Supplementary file 8 [file pcbi.1014224.s006.docx]

# Response to Reviewers

Manuscript: PCOMPBIOL-D-25-00577R3 “Benchmarking Large Language Models for Extracting Biobank-Derived Insights into Health and Disease” Manuel Corpas, Alfredo Iacoangeli. PLOS Computational Biology.

We thank the editor for the opportunity to submit a revised version. This revision addresses, point by point, the issues we disclosed to the handling editor on 21 April 2026, when we identified during proof review that the analysis scripts producing Figures 3 and 4 generated simulated values rather than computing them from the actual model outputs. We have rebuilt those analyses on the empirical data, made the full pipeline reproducible from the public repository, and corrected every affected number and statement in the manuscript. The central conclusion of the study is unchanged: all six models significantly outperform a random baseline (all p < 0.001), confirming genuine biobank-specific knowledge retrieval. The magnitude of that effect is now reported correctly.

The corrected analysis code is public at https://github.com/manuelcorpas/LLM_4_UKB, and every number reported below regenerates deterministically from it.

## Point 1. Figures 3 and 4 were generated from simulated data

Issue: the script that produced Figures 3 and 4 populated the six evaluation dimensions and the random baseline using np.random draws rather than the model outputs, and the headline “16x to 25x improvement over baseline” was an artefact of dividing real coverage by a simulated baseline distribution.

Action: we replaced the script with a pipeline that computes all six dimensions from the same model text responses used for Table 1 (SBERT all-MiniLM-L6-v2 cosine similarity for semantic accuracy; entity matching for factual correctness; concept counting for domain knowledge; structural and interpretive analysis for reasoning quality and response depth; UK Biobank term detection for biobank specificity). The random baseline now draws 10,000 samples per task from the real UK Biobank Schema 19/27 term pools, scored through the identical Weighted Coverage pipeline. Figures 3 and 4 were regenerated from the computed outputs.

## Point 2. Magnitude of improvement over baseline

Issue: the reported 16x to 25x improvement was incorrect.

Action: the empirically computed random baseline mean Weighted Coverage Score is 0.212. The corrected per-model improvement factors are Gemini 3 Pro 3.0x, Claude Sonnet 4.5 2.7x, Claude Opus 4.5 2.7x, Mistral Large 2 2.7x, DeepSeek V3 2.4x, and GPT-5.2 2.1x. The manuscript now states a 2 to 3x improvement throughout (Abstract, Results, Discussion, Figure 4 and its caption).

## Point 3. Statistical test did not match the Methods

Issue: the script implemented a one-sided Z-test while the Methods and the Figure 4 caption described a Mann-Whitney U test.

Action: because each model contributes a single Weighted Coverage Score, a Mann-Whitney U framing is not appropriate (it would require either one observation against the baseline, capping the achievable p, or replicating the single score, which makes the p a function of the replication count). We therefore assess significance with an exact one-sided exceedance test: the proportion of a 10,000-sample random-baseline distribution that reaches or exceeds each model’s score. No baseline sample reached any model’s score, so all six models are significant at p < 0.001 (p < 10^-4). The Methods and Figure 4 caption have been updated to describe this test.

## Point 4. Multidimensional scores

Issue: the per-dimension scores, the consistency index, and the precision/recall values in Figure 3 were simulated.

Action: these are now computed from the model responses. The corrected multidimensional mean scores are Mistral Large 2 0.533, DeepSeek V3 0.518, Gemini 3 Pro 0.452, Claude Opus 4.5 0.436, Claude Sonnet 4.5 0.351, and GPT-5.2 0.286. Per-dimension leaders: semantic accuracy and domain knowledge and biobank specificity, Mistral Large 2; reasoning quality and response depth, DeepSeek V3; factual correctness, Claude Opus 4.5. Claude Sonnet 4.5 shows the highest consistency (0.93). The Results and Discussion text and the Figure 3 caption have been updated to these values, and the previous internal contradictions about which model led each dimension have been resolved.

## Point 5. Model name labels

Issue: two models were mislabelled in the accepted version.

Action: corrected throughout the manuscript, Table 1, and the figures: “Claude Sonnet 4” to “Claude Sonnet 4.5” and “Mistral Large” to “Mistral Large 2”.

## Point 6. Reproducibility of the corrected pipeline

Issue: we wanted to ensure the corrected analysis reproduces exactly, so that the figures in the paper match what any reader obtains from the repository.

Action: we found and fixed a determinism defect in the baseline sampling (the candidate term pools were built from unordered sets, so a fixed random seed did not reproduce the same sample). With the pools deterministically ordered, the pipeline now reproduces byte-for-byte across runs (baseline mean 0.212). A helper script regenerates every manuscript number from the committed result files.

## Point 7. Corpus count corrected (identified during the final reproducibility check)

While verifying that every manuscript number reproduces from the public repository, we identified that the corpus count of “8,549 abstracts” in the previously accepted manuscript did not match the underlying data file, which contains 8,553 publications, 7,932 of which include an abstract. We have corrected the corpus count to 8,553 publications throughout the manuscript (Methods, Results, Discussion, and the Figure 1 caption) and regenerated Figure 1 from the current data file. This was a separate, small error from the figure-simulation issue, but it is corrected as part of the same revision in the interest of complete consistency between the manuscript and the public data. The Schema 27 application count (15,046) was verified and is unchanged.

## What is unaffected

Table 1 (Weighted Coverage Scores), Figure 1 (UK Biobank schema data), and Figure 2 (model benchmark bars) are produced by the original real pipeline (repository scripts 03 to 07) and were not affected by the issue above. Only the model-name labels were corrected in these.

We are grateful for the editor’s handling of this matter and are happy to provide the recomputed analyses, code, or any further detail that would assist.

Manuel Corpas Alfredo Iacoangeli
