# Supplementary material for: Benchmarking large language models for extracting biobank-derived insights into health and disease
Source: PLoS Comput Biol. 2026 Apr 20;22(4):e1014224. doi: 10.1371/journal.pcbi.1014224 (PMC13541248; doi:10.1371/journal.pcbi.1014224)
Supplement: Peer review history

# Peer review: Decision Letter 0

**DOI:** 10.1371/journal.pcbi.1014224.r001

**Authors:** Ilya Ioshikhes, Wei Li

13 May 2025

Large Language Models for Mining Biobank-Derived Insights into Health and Disease

PLOS Computational Biology

Dear Dr. Corpas,

Thank you for submitting your manuscript to PLOS Computational Biology. After careful consideration, we feel that it has merit but does not fully meet PLOS Computational Biology's publication criteria as it currently stands. Therefore, we invite you to submit a revised version of the manuscript that addresses the points raised during the review process.

Please submit your revised manuscript within 60 days Jul 13 2025 11:59PM. If you will need more time than this to complete your revisions, please reply to this message or contact the journal office at ploscompbiol@plos.org. When you're ready to submit your revision, log on to https://www.editorialmanager.com/pcompbiol/ and select the 'Submissions Needing Revision' folder to locate your manuscript file.

Please include the following items when submitting your revised manuscript:

* A rebuttal letter that responds to each point raised by the editor and reviewer(s). You should upload this letter as a separate file labeled 'Response to Reviewers'. This file does not need to include responses to formatting updates and technical items listed in the 'Journal Requirements' section below.

* A marked-up copy of your manuscript that highlights changes made to the original version. You should upload this as a separate file labeled 'Revised Manuscript with Track Changes'.

* An unmarked version of your revised paper without tracked changes. You should upload this as a separate file labeled 'Manuscript'.

If you would like to make changes to your financial disclosure, competing interests statement, or data availability statement, please make these updates within the submission form at the time of resubmission. Guidelines for resubmitting your figure files are available below the reviewer comments at the end of this letter

We look forward to receiving your revised manuscript.

Kind regards,

Wei Li, Ph.D.

Academic Editor

PLOS Computational Biology

Ilya Ioshikhes

Section Editor

PLOS Computational Biology

**Journal Requirements:**

1) We ask that a manuscript source file is provided at Revision. Please upload your manuscript file as a .doc, .docx, .rtf or .tex. If you are providing a .tex file, please upload it under the item type u2018LaTeX Source Fileu2019 and leave your .pdf version as the item type u2018Manuscriptu2019.

2) Please provide an Author Summary. This should appear in your manuscript between the Abstract (if applicable) and the Introduction, and should be 150-200 words long. The aim should be to make your findings accessible to a wide audience that includes both scientists and non-scientists. Sample summaries can be found on our website under Submission Guidelines:

https://journals.plos.org/ploscompbiol/s/submission-guidelines#loc-parts-of-a-submission

3) Please upload all main figures as separate Figure files in .tif or .eps format. For more information about how to convert and format your figure files please see our guidelines:

https://journals.plos.org/ploscompbiol/s/figures

4) We notice that your supplementary information is included in the manuscript file. Please remove them and upload them with the file type 'Supporting Information'. Please ensure that each Supporting Information file has a legend listed in the manuscript after the references list.

5) Please provide a completed 'Competing Interests' statement, including any COIs declared by your co-authors. If you have no competing interests to declare, please state "The authors have declared that no competing interests exist". Otherwise please declare all competing interests beginning with the statement "I have read the journal's policy and the authors of this manuscript have the following competing interests:"

**Reviewers' comments:**

Reviewer's Responses to Questions

Reviewer #1: This manuscript benchmarks several LLMs, such as GPT, Claude, Gemini, Mistral, Llama, and DeepSeek, using UK Biobank-related literature and metadata as ground truth to evaluate their ability to retrieve biomedical insights. The authors assessed model performance using metrics of keyword and coverage, showing each model’s strengths and limitations. My comments are listed below for your consideration.

Strengths:

1. The manuscript addresses a timely topic, exploring the potential of LLMs in handling large, complex biomedical datasets such as the UK Biobank.

2. The methodological approach is clear and systematic, making use of reproducible metrics.

3. Results provide a clear comparative analysis of multiple prominent LLMs.

Limitations:

1. The study appears rather straightforward, primarily testing general-purpose LLMs without additional fine-tuning or adaptation to biobank-specific tasks. This limits the novel contributions significantly, as such general capabilities are relatively well-known.

2. The evaluation relies heavily on keyword coverage metrics. Such metrics do not sufficiently address the depth, accuracy, or semantic relevance of the retrieved information. A more nuanced analysis incorporating precision, recall, and semantic accuracy (beyond basic cosine similarity thresholds) would enhance the robustness of the findings.

3. There is no baseline comparison against simpler random-selection methods. The authors acknowledged this limitation but did not sufficiently justify why such baseline tests were omitted.

4. The study tests a limited set of queries (keywords, top cited papers, authors, institutions), which limits the generalizability and scope of conclusions drawn about the utility of LLMs for complex biomedical data mining tasks. Including a broader variety of queries, such as those requiring deeper inference, hypothesis generation, or interpretation, would better demonstrate the capabilities or limitations of the models.

5. Claims regarding the transformative potential and future routine use of LLMs appear somewhat overstated, given the relatively modest findings of this benchmarking study. The conclusion section could be toned down to align more accurately with the empirical results.

Addressing these points would substantially strengthen the manuscript, potentially making it suitable for reconsideration.

Reviewer #2: This paper benchmarks large language models (LLMs) on answering biobank-related questions. In particular, it evaluated six main LLMs on answering four questions related to the UK Biobank, and the results demonstrate that Gemini 2.0 had the best overall performance. Please see my comments below.

First, these four questions are arguably not the best use cases for LLMs:

What is the subject of the most commonly occurring keywords in UK Biobank papers?

→ This is essentially a topic extraction task, and there is no clear gold standard available. Please see my comment#2 below.

What is the subject of the most cited papers relating to the UK Biobank?

→ Same as 1. No gold standard. Also, LLMs may not be equipped with citation metadata.

Who are the top 20 most prolific authors publishing on the UK Biobank?

→ This is an author retrieval question that can be directly answered by a search engine.

What are the top 10 leading institutions in terms of number of applications to the UK Biobank?

→ Same here. This is a typical retrieval question that can be directly handled by a search engine.

There are no questions evaluating the reasoning or generative capabilities of LLMs, or the impact of retrieval augmentation, which are key use cases for LLMs.

Second, for the subject extraction task, according to the "Ground Truth Results" section, the topics were extracted using tools. For instance, would the topics "female" and "male" also belong to the broader category "human"? Automatically evaluating LLMs based on extracted keywords is not informative. It would be more meaningful to evaluate semantic categories or LLM-generated summaries using manual evaluation on selected samples.

**********

**Have the authors made all data and (if applicable) computational code underlying the findings in their manuscript fully available?**

The PLOS Data policy requires authors to make all data and code underlying the findings described in their manuscript fully available without restriction, with rare exception (please refer to the Data Availability Statement in the manuscript PDF file). The data and code should be provided as part of the manuscript or its supporting information, or deposited to a public repository. For example, in addition to summary statistics, the data points behind means, medians and variance measures should be available. If there are restrictions on publicly sharing data or code —e.g. participant privacy or use of data from a third party—those must be specified.

Reviewer #1: Yes

Reviewer #2: **No:**

**********

PLOS authors have the option to publish the peer review history of their article (what does this mean?). If published, this will include your full peer review and any attached files.

If you choose “no”, your identity will remain anonymous but your review may still be made public.

**Do you want your identity to be public for this peer review?**   For information about this choice, including consent withdrawal, please see our Privacy Policy.

Reviewer #1: No

Reviewer #2: No

[NOTE: If reviewer comments were submitted as an attachment file, they will be attached to this email and accessible via the submission site. Please log into your account, locate the manuscript record, and check for the action link "View Attachments". If this link does not appear, there are no attachment files.]

**Figure resubmission:**

While revising your submission, please upload your figure files to the Preflight Analysis and Conversion Engine (PACE) digital diagnostic tool, https://pacev2.apexcovantage.com/. PACE helps ensure that figures meet PLOS requirements. To use PACE, you must first register as a user. Registration is free. Then, login and navigate to the UPLOAD tab, where you will find detailed instructions on how to use the tool. If you encounter any issues or have any questions when using PACE, please email PLOS at figures@plos.org. Please note that Supporting Information files do not need this step. If there are other versions of figure files still present in your submission file inventory at resubmission, please replace them with the PACE-processed versions.

**Reproducibility:**

?>

# Author comment: Author response to Decision Letter 1

**DOI:** 10.1371/journal.pcbi.1014224.r002

26 Jun 2025

Attachment Submitted filename: Response-to-Reviewers_v1.docx

# Peer review: Decision Letter 1

**DOI:** 10.1371/journal.pcbi.1014224.r003

**Authors:** Ilya Ioshikhes, Wei Li

27 Aug 2025

PCOMPBIOL-D-25-00577R1

Benchmarking Large Language Models for Extracting Biobank-Derived Insights into Health and Disease

PLOS Computational Biology

Dear Dr. Corpas,

Thank you for submitting your manuscript to PLOS Computational Biology. After careful consideration, we feel that it has merit but does not fully meet PLOS Computational Biology's publication criteria as it currently stands. Therefore, we invite you to submit a revised version of the manuscript that addresses the points raised during the review process.

Please submit your revised manuscript within 30 days Oct 27 2025 11:59PM. If you will need more time than this to complete your revisions, please reply to this message or contact the journal office at ploscompbiol@plos.org. When you're ready to submit your revision, log on to https://www.editorialmanager.com/pcompbiol/ and select the 'Submissions Needing Revision' folder to locate your manuscript file.

Please include the following items when submitting your revised manuscript:

* A rebuttal letter that responds to each point raised by the editor and reviewer(s). You should upload this letter as a separate file labeled 'Response to Reviewers'. This file does not need to include responses to formatting updates and technical items listed in the 'Journal Requirements' section below.

* A marked-up copy of your manuscript that highlights changes made to the original version. You should upload this as a separate file labeled 'Revised Manuscript with Track Changes'.

* An unmarked version of your revised paper without tracked changes. You should upload this as a separate file labeled 'Manuscript'.

If you would like to make changes to your financial disclosure, competing interests statement, or data availability statement, please make these updates within the submission form at the time of resubmission. Guidelines for resubmitting your figure files are available below the reviewer comments at the end of this letter.

We look forward to receiving your revised manuscript.

Kind regards,

Wei Li, Ph.D.

Academic Editor

PLOS Computational Biology

Ilya Ioshikhes

Section Editor

PLOS Computational Biology

**Journal Requirements:**

Please upload all main figures as separate Figure files in .tif or .eps format. For more information about how to convert and format your figure files please see our guidelines:

https://journals.plos.org/ploscompbiol/s/figures

**Reviewers' comments:**

Reviewer's Responses to Questions

**Comments to the Authors:**

**Please note here if the review is uploaded as an attachment.**

Reviewer #1: I appreciate the author's efforts in revising this manuscript. It has been greatly improved in technical quality and clarity.

I am satisfied with the revised version, a minor comment is that the newer Gemini 2.5 has been available since March. Given that Gemini 2.0 demonstrated state-of-the-art performance across nearly all benchmarks in this study, the audience will naturally be curious about the performance of its successor. While certainly not a requirement for publication, adding the analysis with Gemini 2.5 would be a timely and impactful addition, offering a more complete and forward-looking perspective.

Reviewer #3: This study evaluates the performance of non-fine-tuned LLMs on 4 specific biobank-related prompts. The work is generally in good shape and contributes to benchmarking efforts in biomedical NLP, but few places need clarification and refinement before the manuscript can be considered for acceptance.

1. As raised in earlier reviews, the study continues to use the same four benchmark questions without clearly explaining why these were selected to represent real-world use cases for UK Biobank–related tasks. While limitations and future directions mention broader tasks, the rationale for at least starting with these particular questions is not clearly justified. A more explicit explanation earlier in the manuscript would help clarify the intended scope/rationale and validate the study design.

2. For the multidimensional evaluation especially dimensions like reasoning quality or response depth, there is insufficient detail on how these were assessed. To ensure transparency and allow reproduction or proper interpretation of results, all scoring procedures should be explicitly described, ideally with rubrics or examples, either in the main text or supporting information.

3. Some figures and descriptions in the results section lack clarity and should be better linked to the methods. For example, Figure 4 does not specify which metric or result is being plotted (is it coverage, weighted coverage, any multidimentional evaluation, or a composite score? Is it about one of the 4 prompt questions, or an aggregation of all?). The text mentions that random baselines were scored using “the same semantic and coverage pipeline,” but it is unclear whether this includes the multidimensional qualitative measures, which may not be meaningful for random outputs. Similarly in Figure 3, it is not clear how the scores are calculated (due to lack of rubric and procedures mentioned in point 2). All analyses in the results section should be clearly traceable to the corresponding methodological steps.

4. It would probably be helpful to explicitly clarify how the proposed benchmark offers value beyond current model comparisons, especially given the rapid evolution of LLMs.

**********

**Have the authors made all data and (if applicable) computational code underlying the findings in their manuscript fully available?**

The PLOS Data policy requires authors to make all data and code underlying the findings described in their manuscript fully available without restriction, with rare exception (please refer to the Data Availability Statement in the manuscript PDF file). The data and code should be provided as part of the manuscript or its supporting information, or deposited to a public repository. For example, in addition to summary statistics, the data points behind means, medians and variance measures should be available. If there are restrictions on publicly sharing data or code —e.g. participant privacy or use of data from a third party—those must be specified.

Reviewer #1: None

Reviewer #3: Yes

**********

PLOS authors have the option to publish the peer review history of their article (what does this mean?). If published, this will include your full peer review and any attached files.

If you choose “no”, your identity will remain anonymous but your review may still be made public.

**Do you want your identity to be public for this peer review?**   For information about this choice, including consent withdrawal, please see our Privacy Policy.

Reviewer #1: No

Reviewer #3: No

[NOTE: If reviewer comments were submitted as an attachment file, they will be attached to this email and accessible via the submission site. Please log into your account, locate the manuscript record, and check for the action link "View Attachments". If this link does not appear, there are no attachment files.]

**Figure resubmission:**

**Reproducibility:**

To enhance the reproducibility of your results, we recommend that authors of applicable studies deposit laboratory protocols in protocols.io, where a protocol can be assigned its own identifier (DOI) such that it can be cited independently in the future. Additionally, PLOS ONE offers an option to publish peer-reviewed clinical study protocols. Read more information on sharing protocols at https://plos.org/protocols?utm_medium=editorial-email&utm_source=authorletters&utm_campaign=protocols

# Author comment: Author response to Decision Letter 2

**DOI:** 10.1371/journal.pcbi.1014224.r004

30 Jan 2026

Attachment Submitted filename: Response-to-Reviewers_R2.docx

# Peer review: Decision Letter 2

**DOI:** 10.1371/journal.pcbi.1014224.r005

**Authors:** Ilya Ioshikhes, Wei Li

3 Mar 2026

PCOMPBIOL-D-25-00577R2

Benchmarking Large Language Models for Extracting Biobank-Derived Insights into Health and Disease

PLOS Computational Biology

Dear Dr. Corpas,

Thank you for submitting your manuscript to PLOS Computational Biology. After careful consideration, we feel that it has merit but does not fully meet PLOS Computational Biology's publication criteria as it currently stands. Therefore, we invite you to submit a revised version of the manuscript that addresses the points raised during the review process.

Please submit your revised manuscript by May 03 2026 11:59PM. If you will need more time than this to complete your revisions, please reply to this message or contact the journal office at ploscompbiol@plos.org. When you're ready to submit your revision, log on to https://www.editorialmanager.com/pcompbiol/ and select the 'Submissions Needing Revision' folder to locate your manuscript file.

Please include the following items when submitting your revised manuscript:

* A letter that responds to each point raised by the editor and reviewer(s). You should upload this letter as a separate file labeled 'Response to Reviewers'. This file does not need to include responses to formatting updates and technical items listed in the 'Journal Requirements' section below.

* A marked-up copy of your manuscript that highlights changes made to the original version. You should upload this as a separate file labeled 'Revised Manuscript with Track Changes'.

* An unmarked version of your revised paper without tracked changes. You should upload this as a separate file labeled 'Manuscript'.

If you would like to make changes to your financial disclosure, competing interests statement, or data availability statement, please make these updates within the submission form at the time of resubmission. Guidelines for resubmitting your figure files are available below the reviewer comments at the end of this letter.

We look forward to receiving your revised manuscript.

Kind regards,

Wei Li, Ph.D.

Academic Editor

PLOS Computational Biology

Ilya Ioshikhes

Section Editor

PLOS Computational Biology

**********

Note: If the reviewer comments include a recommendation to cite specific previously published works, please review and evaluate these publications to determine whether they are relevant and should be cited. There is no requirement to cite these works unless the editor has indicated otherwise.

**Reviewers' comments:**

Reviewer's Responses to Questions

Reviewer #1: I appreciate the efforts the authors have put into this revision. My previous comments have been fully resolved. I am satisfied with the current manuscript and recommend acceptance. Congrats!

Reviewer #3: The authors have addressed most of my concerns. However, my previous Review Point 3 has not been sufficiently addressed. It appears that my original question may have been truncated and reframed in the response. I encourage the authors to revisit the full comment below and respond to it directly:

"Some figures and descriptions in the results section lack clarity and should be better linked to the methods. For example, Figure 4 does not specify which metric or result is being plotted (is it coverage, weighted coverage, any multidimentional evaluation, or a composite score? Is it about one of the 4 prompt questions, or an aggregation of all?). The text mentions that random baselines were scored using “the same semantic and coverage pipeline,” but it is unclear whether this includes the multidimensional qualitative measures, which may not be meaningful for random outputs...."

Specifically, for Figure 4, it remains unclear what exactly the measure of “performance” or “improvement” is being evaluated (given that many benchmark meansures are discussed but only one measure is plotted and a reader would not know what that is), even with the updated caption. Additionally, the manuscript should clarify the rational of using that particular measure of choice (if one exists), especially what does it mean to evaluate it on the null random outputs (e.g. it may not even make sense to evaluate Reasoning Qualuty for random outputs).

The other concerns that I mentioned have been sufficiently addressed, but figures are generally not in publication quality. For example, texts on figure 1 are hard to see.

**********

**Have the authors made all data and (if applicable) computational code underlying the findings in their manuscript fully available?**

The PLOS Data policy requires authors to make all data and code underlying the findings described in their manuscript fully available without restriction, with rare exception (please refer to the Data Availability Statement in the manuscript PDF file). The data and code should be provided as part of the manuscript or its supporting information, or deposited to a public repository. For example, in addition to summary statistics, the data points behind means, medians and variance measures should be available. If there are restrictions on publicly sharing data or code —e.g. participant privacy or use of data from a third party—those must be specified.

Reviewer #1: None

Reviewer #3: Yes

**********

PLOS authors have the option to publish the peer review history of their article (what does this mean?). If published, this will include your full peer review and any attached files.

If you choose “no”, your identity will remain anonymous but your review may still be made public.

**Do you want your identity to be public for this peer review?**   For information about this choice, including consent withdrawal, please see our Privacy Policy.

Reviewer #1: No

Reviewer #3: No

[NOTE: If reviewer comments were submitted as an attachment file, they will be attached to this email and accessible via the submission site. Please log into your account, locate the manuscript record, and check for the action link "View Attachments". If this link does not appear, there are no attachment files.]

**Figure resubmission:**

**Reproducibility:**

To enhance the reproducibility of your results, we recommend that authors of applicable studies deposit laboratory protocols in protocols.io, where a protocol can be assigned its own identifier (DOI) such that it can be cited independently in the future. Additionally, PLOS ONE offers an option to publish peer-reviewed clinical study protocols. Read more information on sharing protocols at https://plos.org/protocols?utm_medium=editorial-email&utm_source=authorletters&utm_campaign=protocols

# Author comment: Author response to Decision Letter 3

**DOI:** 10.1371/journal.pcbi.1014224.r006

31 Mar 2026

Attachment Submitted filename: Response-to-Reviewers_R3.docx

# Editor report: Decision Letter 3

**DOI:** 10.1371/journal.pcbi.1014224.r007

**Authors:** Ilya Ioshikhes, Wei Li

7 May 2026

Benchmarking Large Language Models for Extracting Biobank-Derived Insights into Health and Disease

PLOS Computational Biology

Dear Dr. Corpas,

Thank you for submitting your manuscript to PLOS Computational Biology. After careful consideration, we feel that it has merit but does not fully meet PLOS Computational Biology's publication criteria as it currently stands. Therefore, we invite you to submit a revised version of the manuscript that addresses the points raised during the review process.

Please submit your revised manuscript by Jul 07 2026 11:59PM. If you will need more time than this to complete your revisions, please reply to this message or contact the journal office at ploscompbiol@plos.org. When you're ready to submit your revision, log on to https://www.editorialmanager.com/pcompbiol/ and select the 'Submissions Needing Revision' folder to locate your manuscript file.

Please include the following items when submitting your revised manuscript:

* A letter that responds to each point raised by the editor and reviewer(s). You should upload this letter as a separate file labeled 'Response to Reviewers'. This file does not need to include responses to formatting updates and technical items listed in the 'Journal Requirements' section below.

* A marked-up copy of your manuscript that highlights changes made to the original version. You should upload this as a separate file labeled 'Revised Manuscript with Track Changes'.

* An unmarked version of your revised paper without tracked changes. You should upload this as a separate file labeled 'Manuscript'.

We look forward to receiving your revised manuscript.

Kind regards,

Wei Li, Ph.D.

Academic Editor

PLOS Computational Biology

Ilya Ioshikhes

Section Editor

PLOS Computational Biology

**Reviewers' comments:**

[NOTE: If reviewer comments were submitted as an attachment file, they will be attached to this email and accessible via the submission site. Please log into your account, locate the manuscript record, and check for the action link "View Attachments". If this link does not appear, there are no attachment files.]

**Figure resubmission:**

**Reproducibility:**

?>

# Author comment: Author response to Decision Letter 4

**DOI:** 10.1371/journal.pcbi.1014224.r008

24 Jun 2026

Attachment Submitted filename: Response_to_Reviewers.docx

# Editor report: Decision Letter 4

**DOI:** 10.1371/journal.pcbi.1014224.r009

**Authors:** Ilya Ioshikhes, Wei Li

20 Jul 2026

Dear Dr. Corpas,

We are pleased to inform you that your manuscript 'Benchmarking Large Language Models for Extracting Biobank-Derived Insights into Health and Disease' has been provisionally accepted for publication in PLOS Computational Biology.

Before your manuscript can be formally accepted you will need to complete some formatting changes, which you will receive in a follow up email. A member of our team will be in touch with a set of requests.

Please note that your manuscript will not be scheduled for publication until you have made the required changes, so a swift response is appreciated.

IMPORTANT: The editorial review process is now complete. PLOS will only permit corrections to spelling, formatting or significant scientific errors from this point onwards. Requests for major changes, or any which affect the scientific understanding of your work, will cause delays to the publication date of your manuscript.

Should you, your institution's press office or the journal office choose to press release your paper, you will automatically be opted out of early publication. We ask that you notify us now if you or your institution is planning to press release the article. All press must be co-ordinated with PLOS.

Thank you again for supporting Open Access publishing; we are looking forward to publishing your work in PLOS Computational Biology.

Best regards,

Wei Li, Ph.D.

Academic Editor

PLOS Computational Biology

Ilya Ioshikhes

Section Editor

PLOS Computational Biology

***********************************************************

# Editor report: Acceptance letter

**DOI:** 10.1371/journal.pcbi.1014224.r010

**Authors:** Ilya Ioshikhes, Wei Li

PCOMPBIOL-D-25-00577R3

Benchmarking Large Language Models for Extracting Biobank-Derived Insights into Health and Disease

Dear Dr Corpas,

I am pleased to inform you that your manuscript has been formally accepted for publication in PLOS Computational Biology. Your manuscript is now with our production department and you will be notified of the publication date in due course.

The corresponding author will soon be receiving a typeset proof for review, to ensure errors have not been introduced during production. Please review the PDF proof of your manuscript carefully, as this is the last chance to correct any errors. Please note that major changes, or those which affect the scientific understanding of the work, will likely cause delays to the publication date of your manuscript.

Soon after your final files are uploaded, unless you have opted out, the early version of your manuscript will be published online. The date of the early version will be your article's publication date. The final article will be published to the same URL, and all versions of the paper will be accessible to readers.

For Research, Software, and Methods articles, you will receive an invoice from PLOS for your publication fee after your manuscript has reached the completed accept phase. If you receive an email requesting payment before acceptance or for any other service, this may be a phishing scheme. Learn how to identify phishing emails and protect your accounts at https://explore.plos.org/phishing.

Thank you again for supporting PLOS Computational Biology and open-access publishing. We are looking forward to publishing your work!

With kind regards,

Zsofia Freund

PLOS Computational Biology | Carlyle House, Carlyle Road, Cambridge CB4 3DN | United Kingdom ploscompbiol@plos.org | Phone +44 (0) 1223-442824 | ploscompbiol.org | @PLOSCompBiol
